# A Novel Nature-Inspired Optimization Algorithm: Grizzly Bear Fat Increase Optimizer

**DOI:** 10.3390/biomimetics10060379

**Published:** 2025-06-07

**Authors:** Moslem Dehghani, Mokhtar Aly, Jose Rodriguez, Ehsan Sheybani, Giti Javidi

**Affiliations:** 1 Facultad de Ingeniería, Arquitectura y Diseño, Universidad San Sebastián, Bellavista 7, Santiago 8420524, Chile; dehghani.kau@gmail.com (M.D.); mokhtar.aly@uss.cl (M.A.); 2 School of Information Systems and Management, Muma College of Business, University of South Florida, Tampa, FL 33620, USA; javidi@usf.edu

**Keywords:** optimization, metaheuristic, nature-inspired, benchmark test functions, grizzly bear fat increase optimizer

## Abstract

This paper introduces a novel nature-inspired optimization algorithm called the Grizzly Bear Fat Increase Optimizer (GBFIO). The GBFIO algorithm mimics the natural behavior of grizzly bears as they accumulate body fat in preparation for winter, drawing on their strategies of hunting, fishing, and eating grass, honey, etc. Hence, three mathematical steps are modeled and considered in the GBFIO algorithm to solve the optimization problem: (1) finding food sources (e.g., vegetables, fruits, honey, oysters), based on past experiences and olfactory cues; (2) hunting animals and protecting offspring from predators; and (3) fishing. Thirty-one standard benchmark functions and thirty CEC2017 test benchmark functions are applied to evaluate the performance of the GBFIO, such as unimodal, multimodal of high dimensional, fixed dimensional multimodal, and also the rotated and shifted benchmark functions. In addition, four constrained engineering design problems such as tension/compression spring design, welded beam design, pressure vessel design, and speed reducer design problems have been considered to show the efficiency of the proposed GBFIO algorithm in solving constrained problems. The GBFIO can successfully solve diverse kinds of optimization problems, as shown in the results of optimization of objective functions, especially in high dimension objective functions in comparison to other algorithms. Additionally, the performance of the GBFIO algorithm has been compared with the ability and efficiency of other popular optimization algorithms in finding the solutions. In comparison to other optimization algorithms, the GBFIO algorithm offers yields superior or competitive quasi-optimal solutions relative to other well-known optimization algorithms.

## 1. Introduction

For single or multiple objectives, optimization involves determining the best state for decision/design variables. Prior to the heuristic era of optimization, mathematical problems were solved using analytical approaches. It is necessary for analytical methods to consider derivatives of either the sole objective function or the constraint-penalized objective function, along with values and violations of constraints. Using such data, they are able to find the optimal solution for linear and convex non-linear problems as effectively as possible. The downside to this is that it is vulnerable to entrapment in local optima in complicated issues, those with numerous local optima, and is not available for issues associated with stochastic or indeterminate search spaces. Problems in the real world are characterized by stochastic behaviors and unidentified search spaces. Metaheuristic algorithms were developed as a result. In contrast with derivative-based algorithms, metaheuristic algorithms are derivative-free and do not need any limitations or assumptions. This makes them suitable for solving a wide range of problems [1,2,3,4,5].

There has been an increase in the popularity of metaheuristic algorithms and effective methods used to solve complex optimization problems. There are several factors that drive the popularity. First and foremost, metaheuristic algorithms are very simple. There are fundamental theories or mathematical schemes that are derived from nature, which underlie these metaheuristic schemes. It is generally simple to execute these methods because they are simple and straightforward. Metaheuristics can be applied to real-world problems due to their simplicity. Additionally, current techniques can be used to develop their variants. Secondly, optimization methods are seen as black boxes because they have the ability to provide a group of outputs for a particular problem given a particular group of inputs. These methods can also be easily modified to obtain desirable solutions by altering their parameters and structures. Thirdly, metaheuristic algorithms are characterized by randomness. By exploring the whole search space, metaheuristic algorithms prevent themselves from falling into local optima. Metaheuristics—with complex search spaces or, in particular, with multiple local optima—are made successful by this theory. The flexibility and versatility of these metaheuristics imply their applicability to a wide range of optimization problems, particularly non-differentiable and non-linear problems, as well as complex numerical problems having many local minima. The application of metaheuristic algorithms has been successful in many domains.

There are two types of metaheuristic algorithms: local search algorithms and population-based algorithms. Neighborhood structures are used to enhance local search-based algorithms by taking one solution at a time [6], for example, simulated annealing [7], Variable Neighborhood Search [8], Greedy Randomized Adaptive Search Procedure [9], b-hill climbing [10], Stochastic Local Search [11], tabu searches [12], Guided Local Search [13], and recursive Tabu search [14].

The major benefit of local search-driven algorithms is their rapid search speeds, but their major disadvantage is that they tend to emphasize exploitation over exploration, which increases the risk of becoming trapped in local optima. A population-based algorithm, on the other hand, considers a population at a time, recombining the available solutions and generating a new solution at all iterations. In spite of their effectiveness in detecting useful regions in the search space, the techniques are inefficient at exploiting the searched area [15].

Metaheuristics based on populations generate a group of candidate solutions called ‘populations’. A better set of solutions is generated to replace the initially generated ones. In contrast to single-solution candidates, the new candidates are a collection of solutions rather than a single solution. Some examples of population-based algorithms inspired by nature are the following: Differential Evolution (DE) [16], Particle Swarm Optimization (PSO) [17], Harris Hawk Optimization [18], Grey Wolf Optimizer (GWO) [19], and Whale Optimization Algorithm (WOA) [20].

There are five main categories of population-based metaheuristics, including swarm intelligence-based, evolutionary-based, physics-based, event-based, and plant-based. Based on the principles of biological evolution, evolutionary algorithms evolve iteratively so that they become progressively better as they evolve. Some examples of popular evolutionary algorithms include DE [16], Genetic Algorithm [21], Biogeography-Based Optimizer [22], Evolution Strategy [23], and Evolutionary Programming [24].

Swarm intelligence schemes inspired by the behaviors of bees, wolves, ants, bats, etc. in nature, are the second group. Some examples from this group include PSO [17], Bat Algorithm [25], Crow Search Algorithm [26], Fruit Fly Optimization algorithm [27], Firefly Algorithm [28], Dragonfly Algorithm [29], Ant Lion Optimization [30], GWO [19], Salp Swarm Algorithm [31], Cuckoo Search Algorithm [32], Grasshopper Optimization Algorithm [33], WOA [20], and Dolphin Echolocation [34].

The third group is inspired by the beliefs, lifestyle, and social behaviors of animals and humans, called event-based metaheuristics. Some popular algorithms from this category include Harmony Search [35], Deep Sleep Optimizer [36], Teaching Learning-Based Optimizer (TLBO) [37], Group Search Optimizer [38], and Imperialist Competitive Algorithm [39].

Physics-based algorithms are the fourth category of metaheuristics schemes. Physics-inspired metaheuristics have been developed using laws like gravity, explosions, relativity, and Brownian motion. Some examples in this category are Big Bang Big Crunch [40], Electromagnetic Field Optimization [41], Sine Cosine Algorithm [42], Thermal Exchange Optimization [43], Arithmetic Optimization Algorithm [44], Gravitational Search Algorithm [45], Water Evaporation Optimization [46], Magnetic Charged System Search [47], Central Force Optimization [48], Henry Gas Solubility Optimization [49], Solar System Algorithm [50], and Optics Inspired Optimization [51].

Plant-based algorithms are the fifth category of metaheuristics schemes. Algorithms such as the Tree–Seed Algorithm [52,53], Carnivorous Plant Algorithm (CPA) [54], Improved Carnivorous Plant Algorithm [55], Flower Pollination Algorithm [56], Sunflower Optimization Algorithm [57], Walking Palm Tree Algorithm [58], Bamboo Forest Growth Algorithm [59], Lotus Effect Algorithm [60], and Flower Pollination Algorithm [61] are inspired by biological processes and reproduction mechanisms in plants.

A note should be made about the No Free Lunch (NFL) theorem [62]. As a result of this theorem, all optimization problems cannot be solved efficiently by any metaheuristic. It is also possible for a metaheuristic to perform well on a set of problems while showing inadequate results on a different set of problems. NFL promotes high activity in research in this area, leading to new metaheuristics being proposed yearly and enhancing current approaches. The reason for developing a new metaheuristic is to increase fat in Grizzly bears in order to gain fitness.

Optimization algorithms do not necessarily produce global optimal solutions, which is the key to understanding them. As a result, quasi-optimal solutions to optimization problems can be achieved through the use of optimization algorithms [53,55,63,64,65,66,67,68].

Quasi-optimal solutions are best when they equal the global optimal solutions. If they are not equal, they should be near them. A better algorithm to solve optimization problems is therefore one that can provide a quasi-optimal solution near the global optimal solution when comparing various optimization algorithms. In addition, it is important to keep in mind that although an optimization algorithm may be highly effective at solving a particular optimization problem, it is likely to be ineffective at solving other optimization problems. Thus, many optimization algorithms have been designed to generate quasi-optimal solutions that can be considered more accurate and closer to the global optimal solution.

Optimization algorithm performance is determined by applying them to standard optimization problems that already have a known optimal solution in order to determine how well they perform in providing quasi-optimal results. Optimization algorithms are ranked according to their ability to provide solutions that are as close to the global optimum as possible. This means that new optimization algorithms can always be developed that outperform current algorithms for solving optimization problems.

A new metaheuristic optimization algorithm is described and illustrated in this paper, which is named the Grizzly Bear Fat Increase Optimizer (GBFIO), which mimics the behavior of Grizzly bears to increase fat in their body to survive in winter sleep. The behavior of bears used to design the proposed optimization algorithm was inspired by the authors’ observation of the documentary *Bears*, produced in 2014 by Alastair Fothergill and Keith Scholey [69,70]). There are no previous studies in the optimization literature on this subject to the best of the authors’ knowledge. This work differs from recently published papers in that it simulates a range of bear behaviors, such as finding vegetables, fruits, honey, oysters, and other food resources using memory the sense of smell, hunting animals, and taking care of cubs to avoid being hunted by other animals, and fishing. These behaviors are modeled to incorporate both the advantages of local search-based algorithms and population-based algorithms within a unified framework.

A total of 31 mathematical optimization problems and also 4 constrained engineering design problems such as tension/compression spring design (TCSD), welded beam design (WBD), pressure vessel design (PVD), and Speed reducer design (SRD) problems are solved in this study to appraise the GBFIO algorithm’s effectiveness. The GBFIO’s performance is superior in comparison with state-of-the-art optimization methods based on the outcomes of optimization.

The rest of the paper is organized as follows: the theory of the proposed GBFIO algorithm and its mathematical model is presented in Section 2. In Section 3, 31 mathematical optimization problems and thirty CEC2017 test benchmark functions are presented and solved with the proposed GBFIO algorithm and also compared with four well-known optimization algorithms—PSO, DE, TLBO, and GWO. In Section 4, the proposed GBFIO algorithm is used to solve four engineering problems: TCSD, WBD, PVD, and SRD problems. Finally, the main conclusion is presented in Section 5.

## 2. Grizzly Bear Fat Increase Optimization Algorithm

Firstly, the GBFIO algorithm will be described in this section, and next its mathematical model will be illustrated to optimize diverse optimization problems.

### 2.1. GBFIO Algorithm Theory

**Figure 1:**
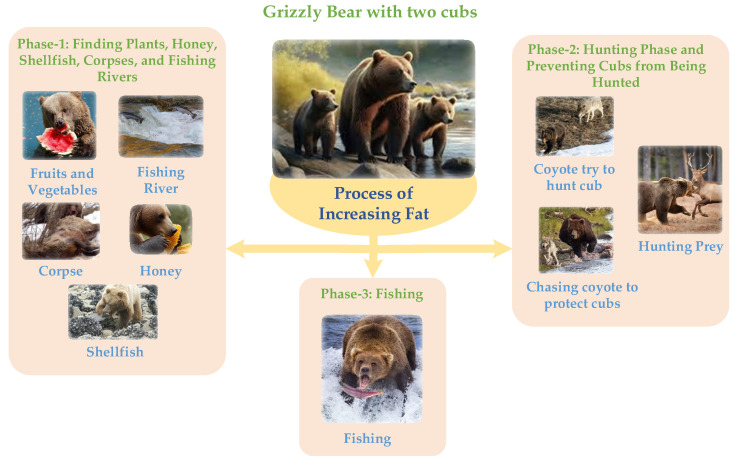
A grizzly bear with two cubs and the process of increasing fat.

Bears must store enough fat in their bodies during the warm months of the year to feed themselves during the cold months when they hibernate and are inactive for several months. Bears with cubs must also store enough fat to nurse their cubs during hibernation, and if they cannot store enough fat, the cubs will die [69,70,71,72]. Figure 1 shows a grizzly bear with her two cubs that is feeding and trying to increase its fat to survive in the winter.

Grizzly bears are omnivores, and their diet depends on the available food sources. In addition to fishing and hunting, brown bears feed on plant materials such as fruits, roots, shellfish, honey, etc. [69,70,71,72]. Therefore, the increase in fat in grizzly bears can be classified into the following three stages, with each step storing some fat in the bear until it reaches the amount needed for hibernation (see Figure 1) [69,70,71,72]: (1) finding the location of vegetables, fruits, shellfish, ponds, rivers for fishing and also following the movement of fish based on the memory of previous years and the sense of smell; (2) hunting other animals and also taking care of the offspring to avoid being hunted; (3) fishing (this is a local search). Therefore, the proposed optimization algorithm based on the increase in fat in grizzly bears is modeled as follows:

#### 2.1.1. Phase 1: Finding Plants, Honey, Shellfish, Corpses, and Fishing Rivers

The main diet of grizzly bears for gaining fat is fish, but until the fish arrive from the sea to the spawning grounds and grizzly bears find a suitable place for fishing, they eat other things, including vegetables, fruits, honey, shellfish, and dead animal carcasses. Therefore, gaining fat by eating vegetables, fruits, shellfish, etc., and also finding fish is modeled as follows:

(1) $$x_{bear}^{search}(t+1)=x_{bear}^{search}(t)+r_{1}.∆x_{fish}+r_{2}.∆x_{honey}+r_{3}.∆x_{shell}+r_{4}.∆x_{corpse}+r_{5}.∆x_{plants},$$

(2) $$∆x_{fish}=x_{fish}-x_{bear}^{search}(t),$$

(3) $$∆x_{honey}=x_{honey}-x_{bear}^{search}(t),$$

(4) $$∆x_{shell}=x_{shell}-x_{bear}^{search}(t),$$

(5) $$∆x_{corpse}=x_{corpse}-x_{bear}^{search}(t),$$

(6) $$∆x_{plants}=x_{plants}-x_{bear}^{search}(t),$$

where $x_{fish}$, $x_{honey}$, $x_{shell}$, $x_{corpse}$, and $x_{plants}$ are fish, honey, shells, corpses, and plants that bears try to find in order to eat and increase the fat for surviving in the winter. $x_{bear}^{search}(t)$ is the current population, and the top five best populations are selected as $x_{fish}$, $x_{honey}$, $x_{shell}$, $x_{corpse}$, and $x_{plants}$, respectively. The current iteration’s number is indicated by t.

Also, as finding each of them is challenging for bears and is found randomly, $r_{1}$ to $r_{5}$ are defined to show the random state of each food source, hence $r_{1}$ to $r_{5}$ are defined as follows:

(7) $$r_{1}^{s}=2.rand.\sqrt{rand},$$

(8) $$r_{2}^{s}=rand.\sqrt{rand},$$

(9) $$r_{3}^{s}=\frac{rand}{2}.\sqrt{rand},$$

(10) $$r_{4}^{s}=\frac{rand}{4}.\sqrt{rand},$$

(11) $$r_{5}^{s}=\frac{rand}{8}.\sqrt{rand},$$

where rand value is selected randomly in the range of [0, 1]. The $r_{1}$ value is larger than other random values because fish is a more interesting food for bears and has a high nutritional value in terms of fat gain. Therefore, with the reduction in nutritional value and its effect on increasing fat, the value of its effect coefficient also decreases, so we have $r_{1}^{s}>r_{2}^{s}>r_{3}^{s}>r_{4}^{s}>r_{5}^{s}$. In addition, the $r_{1}^{s}$, $r_{2}^{s}$, $r_{3}^{s}$, $r_{4}^{s}$, and $r_{5}^{s}$ values are in the range of [0, 2], [0, 1], [0, 0.5], [0. 0.25], [0, 125], respectively.

#### 2.1.2. Phase 2: Hunting Phase and Safeguarding Cubs from Being Hunted

One way that grizzly bears gain fat is by hunting other animals. Female bears must remain vigilant to protect their cubs during the hunt from potential predators, including coyotes and other bears, which affects the hunting process. If the cubs are killed, the bear needs less food and less fat for the winter, which is also modeled. Therefore, the stage of hunting a bear is as follows.

As a first step, bears identify where their prey is and move towards them. As a result of simulating the behavior of the bear, the proposed GBFIO searches the search space in order to discover various search areas. A key feature of the GBFIO is that the prey’s location within the search space is determined at random. Equation (12) simulates how the bear moves to its target and how these concepts work.

(12) $$∆x_{bear}=\left|2.r_{1}^{h}.x_{bear}^{hunt}(t)-r_{2}^{h}.x_{prey}(t)\right|,$$

(13) $$x_{bear}^{hunt}(t+1)=x_{bear}^{hunt}(t)-A.∆x_{bear},$$

where $r_{1}^{h}$, and $r_{2}^{h}$ are randomly selected values in the range [0, 1]. The larger the prey, the more fat the bear accumulates; hence, to have maximize fat accumulation, the best population, obtained after the previous update step, is selected as the prey. So, $x_{bear}^{hunt}$ is the current population that is selected as the bear, which tries to hunt the best prey to increase its fat level. ($x_{prey}(t)$ is the best population which is obtained from the updated population from the previous step). A shows the coefficient vector that has been computed by Equation (14).

(14) $$A=\alpha (2.r_{3}^{h}-1),$$

In which α value is in [0, 2.5], and it decreases linearly from 2.5 to zero within the iteration process, and $r_{3}^{h}$ shows a random value in the range of [0, 1].

In the second step, the predation of the cubs by other animals, including coyotes, is modeled. In this step, it is assumed that the bear has two cubs that she must protect during the hunt and prevent them from being hunted. If the cubs are hunted and killed, the mother bear stores more fat by not feeding the cubs. Hence, the current population, which is the mother bear, can increase more fat if the cubs are hunted by coyote. Three individuals from the population are randomly selected as cubs and coyotes, and also, since the coyote is a predator and it is stronger than cubs, the best member among these three selected members is selected as the coyote, and the other two members are selected as cubs. So, hunting the cubs by the coyote is modeled as follows:

(15) $$\left\{\begin{array}{c} ∆x_{coyote}^{cub-1}=\left|2.r_{4}^{h}.x_{cub}^{\left(1\right)}-r_{5}^{h}.x_{coyote}\right|\\ ∆x_{coyote}^{cub-2}=\left|2.r_{6}^{h}.x_{cub}^{\left(2\right)}-r_{7}^{h}.x_{coyote}\right| \end{array}\right.,$$

(16) $$x_{bear}^{care}\left(t+1\right)=x_{bear}^{care}\left(t\right)-B_{1}.∆x_{coyote}^{cub-1}-B_{2}.∆x_{coyote}^{cub-2},$$

(17) $$B_{1}=B_{2}=\rho (2.r_{8}^{h}-1),$$

where $x_{bear}^{care}\left(t\right)$ is the current population that is selected as the mother bear that should save and increase fat in her body, and $x_{coyote}$, $x_{cub}^{\left(1\right)}$, and $x_{cub}^{\left(2\right)}$ are three random members in the updated population after Phase 1. ($x_{coyote}$ is the best member among these three selected members, and the other two members are chosen as $x_{cub}^{\left(1\right)}$ and $x_{cub}^{\left(2\right)}$). $r_{4}^{h}$, $r_{5}^{h}$, $r_{6}^{h}$, $r_{7}^{h}$, and $r_{8}^{h}$ are the random vectors in [0, 1]. $B_{1}$ and $B_{2}$ show the coefficient vectors that are computed by Equation (17). In which ρ value is in [0, 1.25], and it decreases linearly from 1.25 to zero within the iteration process.

In this section, it is assumed that the bear either hunts and gains fat, or that the bear can store more fat by losing cubs. Since the bear takes care of the cubs and can also survive some attacks by running away and fighting, the bear is more likely to be hunted than the cubs are hunted by coyotes or other bears. Taking the above into account, either the hunting state is considered or the cubs are lost, so we have the following:

(18) $$\left\{\begin{array}{c} x_{bear}^{hunting-care}\left(t+1\right)=x_{bear}^{hunt}\left(t+1\right)\;\;\;\;\;\;if\;\;\;\beta \leq 0.7\\ x_{bear}^{hunting-care}\left(t+1\right)=x_{bear}^{care}\left(t+1\right)\;\;\;\;\;if\;\;\;\beta >0.7 \end{array}\right.,$$

where β is the random value in the range [0, 1].

#### 2.1.3. Phase 3: Fishing

Grizzly bears have a strong preference for fish. Every year, thousands of salmon migrate upstream to spawn. These fish provide the bears with the rich fats and proteins they need to survive. The abundance of fish helps the bears gain the weight they need to survive the winter.

Grizzly bears position themselves along the migratory path of salmon, catching fish as they leap and ascend the river. Each bear occupies a specific location and is capable of catching a certain number of fish per day within a circular fishing area defined by a radius r. With each successful catch, the bear increases the amount of fat in its body. As the cold season progresses and approaches its end, the number of fish decreases and the bear’s fat is closer to the amount needed for hibernation. Therefore, we model the following:

(19) $$x_{bear}^{fishing}\left(t+1\right)=\left(1+F\right).x_{bear}^{fishing}(t),$$

(20) $$F=\eta .\gamma .cos(2.\pi .r_{1}^{f}),$$

where $x_{bear}^{fishing}(t)$ is the current updated population after phase 2, corresponding to bears engaged in fishing to increase their fat reserves. The term $cos(2.\pi .r_{1}^{f})$ is used to model the circular fishing area, where $r_{1}^{f}$ is the random value in [0, 1]. The parameter γ is in [0, 1], and it denotes a decay factor that decreases linearly from 1 to 0 within the iteration process. The constant η is set at 0.3.

An adult grizzly bear catches about 25 fish per day. To account for the daily fish catch, $f^{th}\;$ is the number of fishing attempts (or repetitions) per day for each bear to increase its fat. Therefore, we express the fishing phase as follows:

(21) $${\left(x_{bear}^{fishing}\left(t+1\right)\right)}^{\left(f\right)}={\left(\left(1+F\right).x_{bear}^{fishing}\left(t\right)\right)}^{\left(f\right)},\;\;\;\;\;\;\;\;\forall \;\;f=\{1,\;2,\ldots ,\;25\},$$

where the maximum number of f equals 25. In general, in the fishing phase, each population is updated 25 times around its current position in each iteration. After each fishing attempt, if the newly generated solution (position) yields a better fitness value, it replaces the previous one.

### 2.2. Pseudo-Code and Execution Procedure of the Proposed GBFIO Algorithm

In this section, the execution procedure of the proposed GBFIO algorithm is presented step by step as follows:

Step 1: Set input values including the number of variables (n), maximum and minimum values of variables, maximum iteration number ($Iter_{max}$), and the population size (N). Initialize the suggested GBFIO algorithm parameters including fishing number (f = 25) and η=0.3. Finally, set the maximum number of the following parameters: α=2.5, ρ=1.25, γ=1.

Step 2: Generate the initial population randomly within the range of variable values as follows:

(22) $${\bar{X}}_{pop}=rand(1,n)*\left(X_{max}-X_{min}\right)+X_{min},$$

where $X_{max}$ and $X_{min}$ are the minimum and maximum values of the variables. rand shows a random vector in [0, 1]. Also, the objective function for each population is computed. So that the primary population can be computed, this procedure has been repeated for each parameter.

Step 3: Start the iteration and set iteration=1+iteration.

Step 4: Start the searching phase by selecting the top 5 best members based on objective functions such as fish, honey, shells, corpses, and plants, respectively. Compute the entire new searching population based on Equations (1)–(11) (each population is selected as a bear searching for food).

Step 5: Compute the objective function for the entire new computed population in Step 4. If the objective function of each new searching population is better than the initial population, then update the initial population by the new searching population that is computed in Step 4.

Step 6: Start the Hunt and Care Phases.

Step 6.1—Hunt Phase: Select the best-performing member of the population as the prey, which will provide the most fat for the bears. Compute the entire new hunting population based on Equations (12)–(14).

Step 6.2—Care Phase: Select three random members of the population. Among them, identify the best-performing one as the coyote and the other two as bear cubs. Compute the entire new care population based on Equations (15)–(17).

Step 6.3—Select the new population of the Hunt and Care Phases based on Equation (18). If the generated random number is lower than 0.7, then the new hunt and care population ($x_{bear}^{hunting-care}$) is selected from the hunting phase (Step 6.1); if the generated random number is more than 0.7, then the new Hunt and Care population ($x_{bear}^{hunting-care}$) is selected from the care phase (Step 6.2).

Step 7: Compute the objective function for the entire new computed population in Step 6.3. If the objective function of each new Hunt and Care population ($x_{bear}^{hunting-care}$) is better than the updated initial population, then replace the population with the new Hunt and Care population ($x_{bear}^{hunting-care}$) that is computed in Step 6.

Step 8: Start the fishing phase. Each updated population represents a bear that performs fishing 25 times each day. Set the fishing number as f=1.

Step 8.1: Compute the new fishing population by Equation (21) and then compute the objective function.

Step 8.2: If the objective function of each fishing population ($x_{bear}^{fishing}$) is better than the updated initial population, then replace the population with the new fishing population ($x_{bear}^{fishing}$).

Step 8.3: If the number of fishing times is less than the maximum fishing number (25), then increment the fishing counter f = f + 1 and return to Step 8.1. Otherwise, go to Step 9.

Step 9: If the number of iteration is less than the maximum number of iteration, then go to Step 4; otherwise, go to Step 10.

Step 10: Select the best member of the updated population as a solution and then end the procedure.

**Figure 2:**
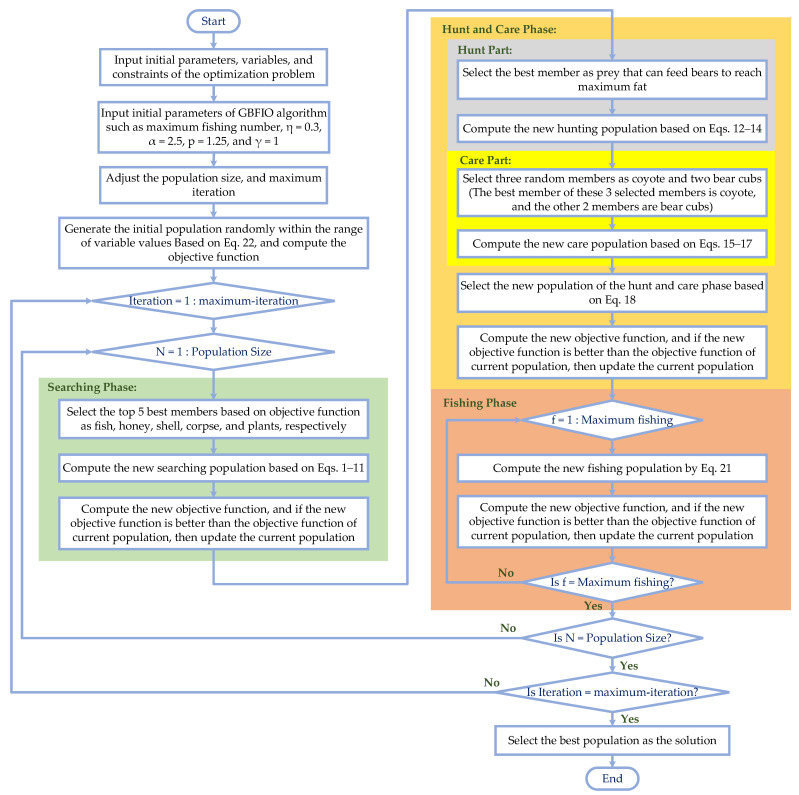
Flowchart of the suggested GBFIO algorithm.

The pseudo-code of the proposed GBFIO algorithm is described in Table 1. In addition, the flowchart of the GBFIO algorithm is shown in Figure 2.

## 3. Results and Discussion

### 3.1. Benchmark Functions

There are several experiments carried out to evaluate the suggested algorithm’s effectiveness and provide numerical evidence for those theoretical claims. In total, 31 benchmark functions have been applied to assay the GBFIO; 23 of the first test functions correspond to classical benchmark functions. They are employed in a variety of types [73].

As shown in Table 2, Table 3, Table 4 and Table 5, the benchmark functions are categorized into three groups: unimodal benchmark (UB), high-dimensional multimodal (HDMM), and fixed-dimensional multimodal (FDMM), as well as rotated and shifted benchmark (RSB) functions.

The UB functions with a single optimum can be applied to test the exploitation abilities of optimization algorithms. As shown in Table 2, there are seven fixed-dimension and scalable UB functions ($CF_{1}$ to $CF_{7}$).

The multimodal benchmark functions present greater challenges compared to UB functions because they contain more than one minimum. Multimodal benchmark functions have been applied to test the exploration and evasion of local minima in optimizers. The 16 HDMM and FDMM test functions used to test the GBFIO ($CF_{8}$ to $CF_{23}$) are listed in Table 3 and Table 4.

In the final group, the RSB functions exhibit higher complexity and follow the composition function paradigm [74]. Table 5 shows the mathematical models for each benchmark function ($CF_{24}$ to $CF_{31}$).

These tables are arranged by dimension to indicate the size of the problem,$\;F_{min}$ to indicate the minimum found in the research, and Range to indicate the boundaries of the problem’s search space.

### 3.2. Results

In this case, the performance of the proposed GBFIO in solving optimization problems is investigated. Hence, 31 objective functions of diverse kinds of unimodal, HDMM, FDMM, and RSB functions have been solved by using the GBFIO. Table 2, Table 3, Table 4 and Table 5 show the used benchmark functions’ details. Additionally, a comparison is also made between the proposed GBFIO optimization algorithm and PSO, DE, TLBO, and GWO, four popular algorithms. In order to implement the objective functions, 500 independent implementations having 500 iterations were implemented. The number of populations selected is 150. The parameter values of the proposed optimization algorithms are expressed as follows: GBFIO (f = 2.5; eta = 1.25; Zeta = 0.3; fishing_number = 25), PSO (Cognitive constant, $C_{1}$ = 2; Social constant, $C_{2}=2$; Local constant; W is linearly reduced from 0.9 to 0.1), DE (Crossover rate, $c_{r}=0.7$; Mutation coefficient, F = 0.2), TLBO (Teaching factor F is randomly selected between [1, 2]).

These tests are run on a Windows 10 Pro system using Intel Core i7-4600U, 2.1~2.7 GHz, 12G RAM and, Matlab R2019a. The algorithms were run 20 times on each mentioned benchmark function. The statistical outcomes (Average (Ave), Standard Deviation (SD), and rank of each algorithm in comparison to each other (Rank-1 is defined based on the average solution, and Rank-2 is defined according to the best solution achieved in 20 independent runs) have been expressed in Table 6, Table 7, Table 8 and Table 9.

Mean and SD indexes are useful to demonstrate algorithms’ ability to avoid local minima. As the Mean index decreases, the algorithm becomes more likely to find a solution that is close to the global optimum. Despite equal Mean values, each algorithm may perform differently in determining the global optimum. As a result, SD allows for a more accurate comparative analysis. A small SD will result in a lower degree of dispersion of results.

There are three criteria for reporting simulation outcomes: (i) the average of the optimal solutions achieved (Mean), (ii) the standard deviation of the optimal solutions achieved (SD), and (iii) the optimal candidate solution (Best). Equations (23) and (9) can be used to calculate Mean and SD.

(23) $$Mean=\frac{1}{N_{i}}\sum_{l=1}^{N_{i}}BCF_{l},$$

(24) $$SD=\sqrt{\frac{1}{N_{i}}\sum_{l=1}^{N_{i}}{\left(BCF_{l}-Mean\right)}^{2}},$$

In which, $N_{i}$ defines the number of independent runs and $BCF_{l}$ shows the best candidate solution acquired in the $l^{th}$ independent run.

#### 3.2.1. Assessment of UB Functions

**Figure 3:**
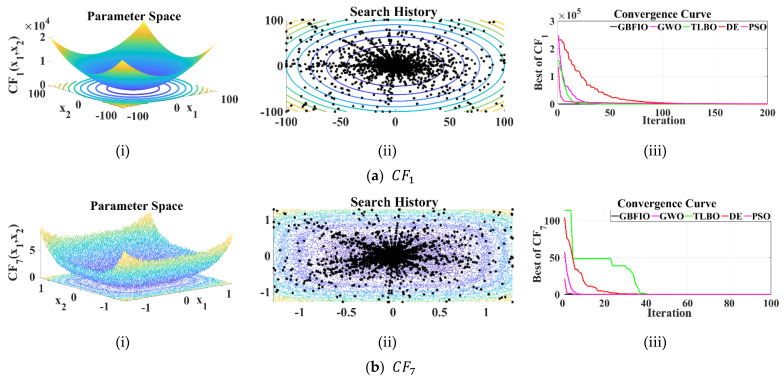
Results of selected UB functions (100-dimensional): (**i**) 2D versions of UB functions; (**ii**) search history; (**iii**) convergence curves.

According to Table 2, $CF_{1}$ to $CF_{7}$ are unimodal objective functions. A comparison of the proposed GBFIO algorithm compared to four rival algorithms is performed on these functions. A comparison of the $CF_{1}$ to $CF_{7}$ functions is given in Table 6. Based on the table, the suggested algorithm for the $CF_{6}$ with 100 dimension would converge to zero, which is the global optimal. The GBFIO is also the most efficient optimizer for the $CF_{1}$, $CF_{2}$, $CF_{3}$, $CF_{4}$, $CF_{5}$, and $CF_{7}$ functions. Comparisons of optimization algorithms show that the GBFIO produces considerably better outcomes than its competitors and is nearer the global optimal. Figure 3 depicts the 2D versions of selected UB functions, the search history, and the convergence curve for the 100-dimensional version of the UB functions for the proposed GBFIO algorithm in comparison to other selected algorithms.

#### 3.2.2. Assessment of HDMM Functions

The proposed GBFIO and other algorithms are analyzed and considered in solving HDMM functions. Six objective functions, $CF_{8}$ to $CF_{13}$, are chosen, which is described in Table 3. The results of the run of the GBFIO and other optimization algorithms for HDMM functions are described in Table 7. Based on the GBFIO proposal, the global optimal has been found for $CF_{9}$ to $CF_{13}$ with zero convergence. The proposed algorithm comes out on top as the best algorithm in terms of quasi-optimal solutions for $CF_{8}$ to $CF_{13}$, except $CF_{12}$, which achieved the second rank, and also the solution is very close to zero. DE is the best algorithm for $CF_{12}$ while the solution of the GBFIO is very close to the solution of DE. According to the simulation results, it is observed that the proposed GBFIO algorithm has high ability and proficiency in solving this kind of optimization problem.

**Figure 4:**
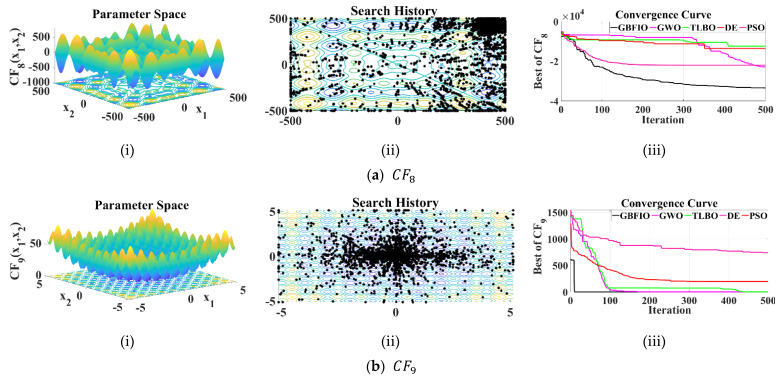
Results of the selected HDMM (100-Dimensional): (**i**) 2D versions of UB functions; (**ii**) search history; (**iii**) convergence curves.

Figure 4 depicts the 2D versions of selected HDMM functions, the search history, and the convergence curve for the 100-dimensional version of the HDMM functions for the suggested GBFIO algorithm in comparison to other selected algorithms.

#### 3.2.3. Assessment of FDMM Functions

**Figure 5:**
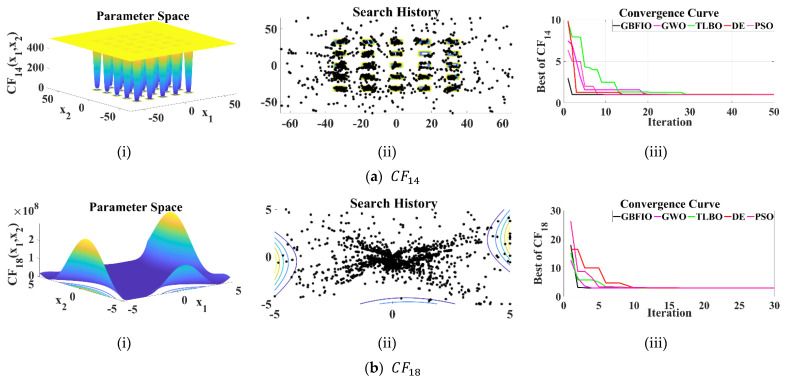
Results of the selected FDMM: (**i**) 2D versions of UB functions; (**ii**) search history; (**iii**) convergence curves.

As shown in Table 4, $CF_{14}$ to $CF_{23}$ evaluate the ability of optimization algorithms to deal with FDMM problems. Table 8 shows optimization outcomes for each objective function using the GBFIO and four competitors’ methods. The GBFIO is shown to converge to the global optimal for $CF_{14}$ to $CF_{23}$. The GBFIO is also the most efficient optimizer (based mean of best solutions) for solving $CF_{14}$ to $CF_{18}$, $CF_{20}$, and $CF_{21}$. (For $CF_{19}$ and $CF_{23}$, the GBFIO algorithm converges to the solution and only based on the SD criterion, it achieves the second rank). Consequently, the proposed GBFIO solves the objective functions more efficiently. Simulation outcomes shown that the GBFIO performed better than the four rival algorithms in solving FDMM optimization problems. Figure 5 depicts the 2D versions of chosen FDMM functions, the search history, and the convergence curve for the proposed GBFIO algorithm in comparison to other selected algorithms.

#### 3.2.4. Assessment of RSB Functions

A detailed evaluation will be conducted here by using recently introduced RSB functions presented in Table 5 [73,74]. Table 5 describes benchmark functions from $CF_{24}$ to $CF_{31}$ that follow a composition paradigm. Table 9 shows the GBFIO and four rival methods’ outcomes to optimize these objective functions. It has been shown that the GBFIO can converge close to the global optimal for $CF_{24}$ to $CF_{31}$. The GBFIO is the top best optimizer (based mean of best solutions) in solving $CF_{26}$, $CF_{27}$, $CF_{29}$, and $CF_{31}$. (For $CF_{24}$ and $CF_{25}$, the GBFIO algorithm converges to the solution and only based on the SD criterion, it achieves another rank). Furthermore, the DE algorithm takes the first rank for $CF_{28}$ and $CF_{30}$, while the solution of GBFIO convergence to the solution. Consequently, the suggested GBFIO solves the objective functions more efficiently. Simulation outcomes shown that GBFIO performed better than the four rival algorithms in solving RSB optimization problems.

**Figure 6:**
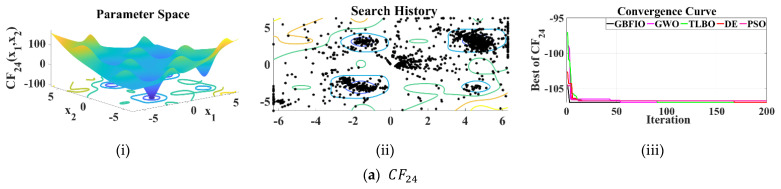

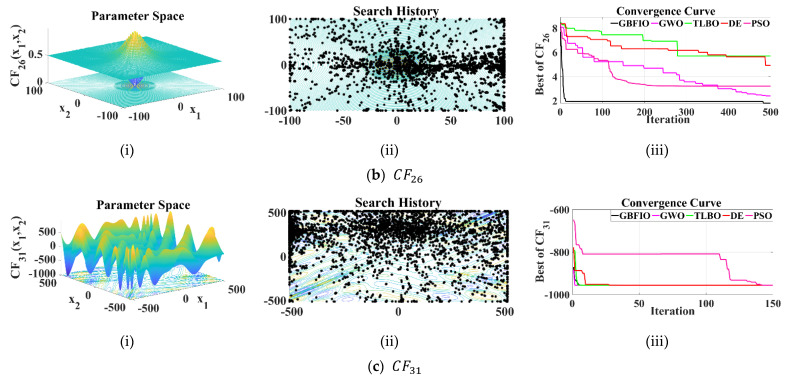
Results of the selected RSB functions: (**i**) 2D versions of UB functions; (**ii**) search history; (**iii**) convergence curves.

Figure 6 depicts the 2D versions of selected RSB functions, the search history, and the convergence curve for the proposed GBFIO algorithm in comparison to other selected algorithms.

### 3.3. Statistical Analysis

The Mean and SD indexes can be used to compare and evaluate optimization techniques based on the optimization results of objective functions. However, it is still possible that one algorithm may be better than others based on randomness, even after a number of different executions. The following subsection presents a statistical analysis known as a Wilcoxon sum rank test [75] to demonstrate to what extent the GBFIO is superior to four rival algorithms. The Wilcoxon sum rank test measures similarities between two dependent samples using a non-parametric statistical method. Differences between the two samples are tested to determine whether or not they are statistically important. An indicator known as a *p*-value is used in the Wilcoxon sum rank test for determining the statistical significance of differences between two algorithms used to optimize various objective functions. Table 10 presents the outcomes of the simulation for the proposed GBFIO against four rival algorithms. The table shows that a *p*-value below 0.05 indicates that the proposed GBFIO is significantly superior to the rival algorithm for each objective function.

### 3.4. Comparison-Based Achieved Rank

**Figure 7:**
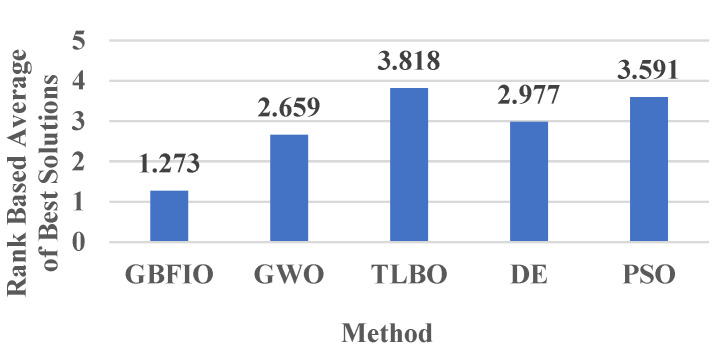
Rank of optimization methods based on the average of best solutions.

**Figure 8:**
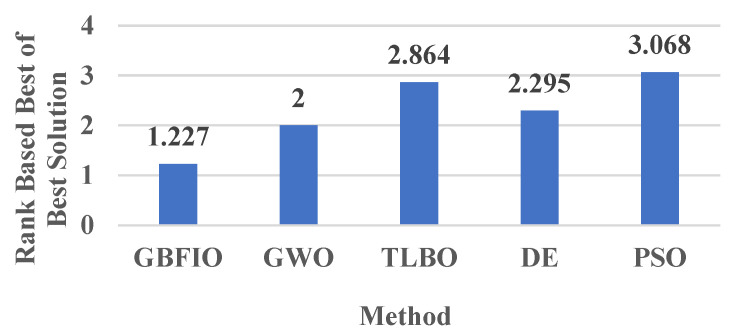
Ranking of optimization methods based on the best of the best solution.

Here, the proposed GBFIO is compared with GWO, TLBO, DE, and PSO based on the achieved ranks between one another in the average of the best solution and also the best of the best solution. The average values of the mentioned ranks in Table 6, Table 7, Table 8 and Table 9 are computed for this comparison. Figure 7 depicts the rank of optimization algorithms according to the average of the best solution rank, which is achieved for 31 test functions. Figure 8 depicts the rank of optimization algorithms based on the best of the best solution rank, which is achieved for 31 test functions. As can be seen, the GBFIO algorithm achieved the best average ranks (1.273 and 1.227) in this comparison. Hence, the proposed GBFIO is more efficient compared to other algorithms to solve the mentioned 31 test functions. By observing the outcomes, it can be concluded that the suggested GBFIO has high ability and capability in solving the optimization problem.

### 3.5. Sensitivity Analysis

The purpose of the following subsection is to introduce sensitivity evaluations of the proposed GBFIO for two parameters: the population size and the maximum number of iterations.

All thirty-one objective functions for various population sizes with 20, 30, 40, 50, and 60 members are subject to a sensitivity assessment of the GBFIO’s efficiency to population sizes (maximum iteration is 500). Table 11 presents the results of the GBFIO under various population sizes that are obtained in 20 independent runs. As can be seen in Table 11, the proposed GBFIO tends to converge to more appropriate quasi-optimal solutions as population sizes increase, resulting in a decrease in the objective function values as the number of members increases.

The suggested algorithm was tested on all thirty-one objective functions to examine the performance’s sensitivity to the maximum number of iterations, which is selected as 50, 100, 200, 500, and 1000 iterations (population size is 80). The assessment outcomes for different maximum number of iterations of the 20 independent runs are presented in Table 12. As can be seen in Table 12, by increasing the number of iterations, the proposed GBFIO leads to solutions that are closer to the global optimal based on the sensitivity assessment.

### 3.6. Comparison-Based Maximum Number of Objective Function Calculation Times

To have a fair comparison, in this sub-section, the GBFIO is compared to four well-known algorithms, GWO, TLBO, DE, and PSO, with maximum 250,000 objective function calculation times. The results of the comparison are presented in Table 13. As can be seen, the suggested GBFIO algorithm reached the first total rank in the best of the best solution and average of the best solution in 20 independent runs.

### 3.7. Comparison of the Proposed GBFIO Algorithm with Other Optimization Algorithms for Shifted and Rotated Unconstrained CEC2017 Test Functions

In this sub-section, to consider the performance of the proposed GBFIO algorithm under CEC2017 test functions, the GBFIO is compared to seven optimization algorithms, GWO, TLBO, DE, PSO, CPA, Tunicate Swarm Algorithm (TSA) [76], and WOA, with maximum 300,000 objective function calculation times for 10 independent runs in the same conditions.

As shown in Table 14, the CEC2017 benchmark test functions include thirty functions such as three shifted and rotated (S&R) unimodal, seven S&R multimodal, ten S&R hybrid and ten composition functions [53,55,59]. For the entire thirty CEC2017 functions, the dimension is considered as thirty, and the range is [−100, 100].

The results of comparison on CEC2017 test functions are presented in Table 15. As can be seen, the suggested GBFIO algorithm reached the first total rank in the best of the best solutions and average of the best solutions in 10 independent runs.

## 4. GBFIO in Engineering Problem

These sections investigate the algorithm’s efficiency using four engineering design problems. A comparison of the GBFIO algorithm and four popular algorithms, GWO, TLBO, DE, and PSO, is also conducted to verify the outcomes.

The following section uses four constrained engineering design problems: TCSD, WBD, PVD, and SRD. Due to the different inequality and equality constrained in these problems, the GBFIO must also have a constrained handling strategy for optimizing constrained problems. Therefore, in the GBFIO, the easiest constraint handling strategy, penalty functions, is used efficiently to handle constraints. In the case of a violation of all constraints, the search agents will receive big objective function values. The simple, scalar penalty functions are used for the problems.

### 4.1. TCSD Problem

**Figure 9:**
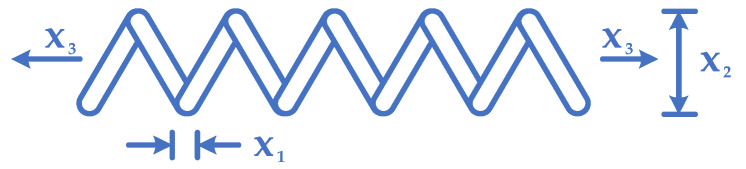
Schematic of TCSD.

The optimization considers a spring construction that acts under tension and compression from the load illustrated in Figure 9 [20,77,78]. A spring volume weight reacting to tension or compression under a load should be minimized. The design variables are $x_{1}$, $x_{2}$, and $x_{3}$ representing the number of active coils, winding diameter, and wire diameter, respectively. Optimization is accomplished by optimizing a spring volume described as follows:
Minimize$f\left(\vec{x}\right)=x_{1}^{2}x_{2}(x_{3}+2),$
Subject to:$\left\{\begin{array}{c} \begin{array}{c} g_{1}\left(\vec{x}\right)=1-\frac{x_{2}^{3}x_{3}}{71785x_{1}^{4}}\leq 0\\ g_{2}\left(\vec{x}\right)=\frac{4x_{2}^{2}-x_{1}x_{2}}{12566(x_{2}x_{1}^{3}-x_{1}^{4})}+\frac{1}{5108x_{1}^{2}}-1\leq 0 \end{array}\\ \begin{array}{c} g_{3}\left(\vec{x}\right)=1-\frac{140.45x_{1}}{x_{2}^{2}x_{3}}\leq 0\\ g_{4}\left(\vec{x}\right)=\frac{x_{1}+x_{2}}{1.5}-1\leq 0 \end{array} \end{array}\right.$(25)Variable range:$\left\{\begin{array}{c} 0.05\leq x_{1}\leq 2.0\\ 0.25\leq x_{2}\leq 1.3\\ 2.00\leq x_{3}\leq 15.0 \end{array}\right.,$


**Figure 10:**
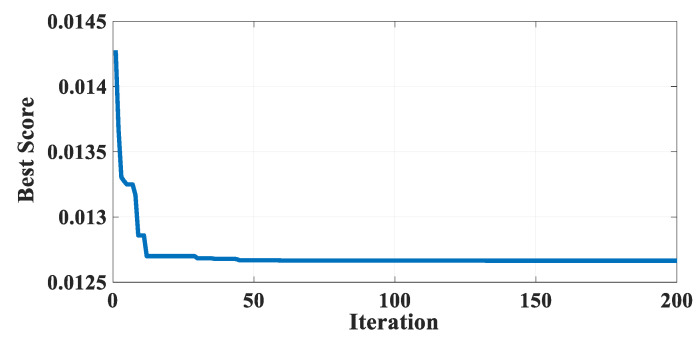
Convergence curve of the GBFIO on the TCSD problem.

Table 16 describes the results of GBFIO, GWO, TLBO, DE, and PSO algorithms, which achieved the best solution for TCSD variables. The outcomes show that GBFIO, DE, and PSO achieve the best solution for the TCSD problem with the objective function equals to 0.012665. Statistical results of the GBFIO algorithm in comparison to other algorithms on the TCSD problem are shown in Table 17, which shows the GBFIO boasts better values for statistical indicators than its competitors. Figure 10 shows the GBFIO convergence curve achieves optimal values for the TCSD problem. A similar penalty function is used for the GBFIO to make accurate comparisons.

### 4.2. WBD Problem

**Figure 11:**
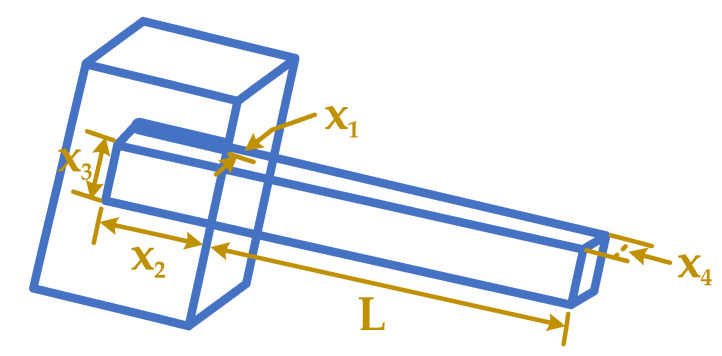
Schematic of WBD.

A construction consisting of two parts, beam and weld, is considered as an optimization problem as shown in Figure 11 [19,79,80]. The model variables show the dimensions of a welded part, that is optimized for minimizing the weld’s cost and the beam’s material without deviating during optimization. There are four variables in the problem: weld thickness ($x_{1}$), attached part of the bar’s length ($x_{2}$), bar height ($x_{3}$), and bar thickness ($x_{4}$). Here are the mathematical formulas:
Minimize$f\left(\vec{x}\right)=1.10471x_{1}^{2}x_{2}+(4.811\times 10^{-2})\times x_{3}x_{4}(14+x_{2}),$
Subject to:$\left\{\begin{array}{c} \begin{array}{c} g_{1}\left(\vec{x}\right)=\tau \left(x\right)-\tau_{max}\leq 0\\ \begin{array}{c} g_{2}\left(\vec{x}\right)=\sigma \left(x\right)-\sigma_{max}\leq 0\\ g_{3}\left(\vec{x}\right)=\delta \left(x\right)-\delta_{max}\leq 0 \end{array} \end{array}\\ \begin{array}{c} \begin{array}{c} g_{4}\left(\vec{x}\right)=x_{1}-x_{4}\leq 0\\ g_{5}\left(\vec{x}\right)=P-P_{c}(x)\leq 0 \end{array}\\ \begin{array}{c} g_{6}\left(\vec{x}\right)=0.125-x_{1}\leq 0\\ g_{7}\left(\vec{x}\right)={1.10471x}_{1}^{2}+(4.811\times 10^{-2}){\times x}_{3}x_{4}(14+x_{2})-5\leq 0 \end{array} \end{array} \end{array}\right.$(26)Variable range:$\left\{\begin{array}{c} \begin{array}{c} 0.1\leq x_{1}\leq 2.0\\ 0.1\leq x_{2}\leq 10 \end{array}\\ \begin{array}{c} 0.1\leq x_{3}\leq 10\\ 0.1\leq x_{4}\leq 2.0 \end{array} \end{array}\right.,$

where τ, δ, σ, and $P_{c}$ are represented the shear stress, end deflection of the beam, bending stress in the beam and buckling load on the bar, respectively. In addition, the other parameters are in the following:

(27) P=6000 lbL=14 inE=30×106 psiG=12×106 psiτmax=13600 psiσmax=3×104 psiδmax=0.25 in,τx=τ′2+2τ′τ″x22R+τ″2τ′=P2x1x2τ″=MRJM=P(L+x22)R=x224+x1+x222σx=6PLx32x4δx=4PL2Ex32x4J=2{2x1x2[x2212+x1+x322]}Pcx=4.013Ex32x4636L2(1−x32LE4G)

**Figure 12:**
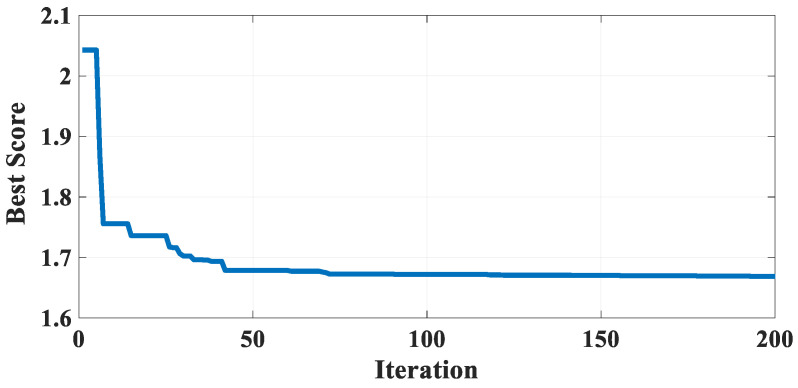
Convergence curve of the GBFIO on the WBD problem.

Table 18 describes the results of GBFIO, GWO, TLBO, DE, and PSO algorithms, which achieved the best solution for WBD variables. The outcomes show that GBFIO, DE, and PSO achieve the best solution for the WBD problem, with the objective function equalling 1.668085. Statistical results of the GBFIO algorithm in comparison to other algorithms on the WBD problem are shown in Table 19, which shows that the GBFIO boasts better values for statistical indicators than its competitors. Figure 12 shows the GBFIO convergence curve that achieves optimal values for the WBD problem. A similar penalty function is used for the GBFIO to make accurate comparisons.

### 4.3. PVD Problem

**Figure 13:**
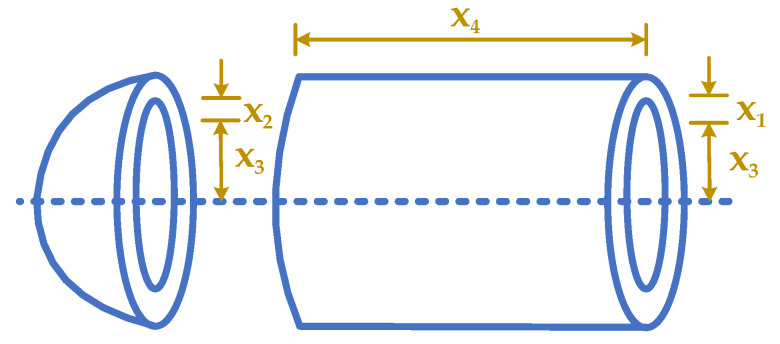
Schematic of PVD.

As can be seen in Figure 13, the optimization problem considers a gas storage container with two hemispherical cylinders at either end [19,79,80]. Containers like this are employed for liquid gases that need to be kept under pressure to maintain their features. The maximum pressure of 1000 [psi] for the minimal volume of 750 [$ft^{3}$] should be kept by optimizing the construction weight of the object. Four variables which are in the PVD optimization problem: the shell thickness ($x_{1}$), the head thickness ($x_{2}$), the inner radius ($x_{3}$), and the cylindrical section length excluding the head ($x_{4}$). The mathematical formulation of the PVD problem is described below:
Minimize$f\left(\vec{x}\right)=0.6224x_{1}x_{3}x_{4}+1.7781x_{2}x_{3}^{2}+3.1661x_{1}^{2}x_{4}+19.84x_{1}^{2}x_{3},$
Subject to:$\left\{\begin{array}{c} \begin{array}{c} g_{1}\left(\vec{x}\right)=-x_{1}+0.0193x_{3}\leq 0\\ g_{2}\left(\vec{x}\right)=-x_{2}+0.00954x_{3}\leq 0 \end{array}\\ \begin{array}{c} g_{3}\left(\vec{x}\right)=1296000-\frac{4}{3}\pi x_{3}^{3}-\pi x_{3}^{2}x_{4}\leq 0\\ g_{4}\left(\vec{x}\right)=x_{4}-240\leq 0 \end{array} \end{array}\right.$(28)Variable range:$\left\{\begin{array}{c} \begin{array}{c} 0\leq x_{1}\leq 99\\ 0\leq x_{2}\leq 99 \end{array}\\ \begin{array}{c} 10\leq x_{3}\leq 200\\ 10\leq x_{4}\leq 200 \end{array} \end{array}\right.$


**Figure 14:**
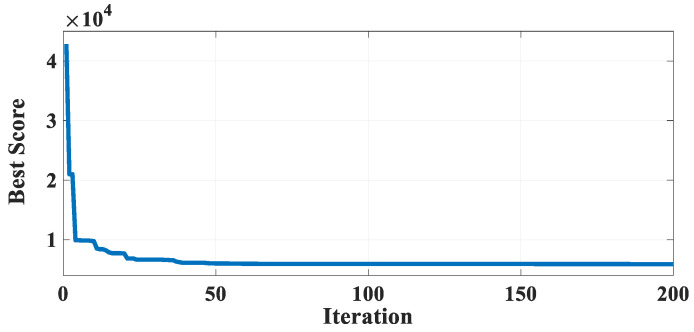
Convergence curve of the GBFIO on the PVD problem.

Table 20 presents the results of GBFIO, GWO, TLBO, DE, and PSO algorithms, which achieved the best solution for PVD variables. The outcomes show that PSO and GBFIO algorithms achieve the first and second ranks in finding the best solution for the PVD problem, respectively. As can be seen, GBFIO achieved the second rank with a small difference; as shown in Table 21, the GBFIO algorithm has a low SD, highlighting its robustness and consistency in finding optimal solutions. Statistical results of the GBFIO algorithm in comparison with other algorithms on the PVD problem are shown in Table 21, which shows the GBFIO algorithm boasts better values for statistical indicators than its competitors. Figure 14 shows the GBFIO convergence curve that achieves optimal values for the PVD problem. A similar penalty function is used for GBFIO to make accurate comparisons.

### 4.4. SRD Problem

**Figure 15:**
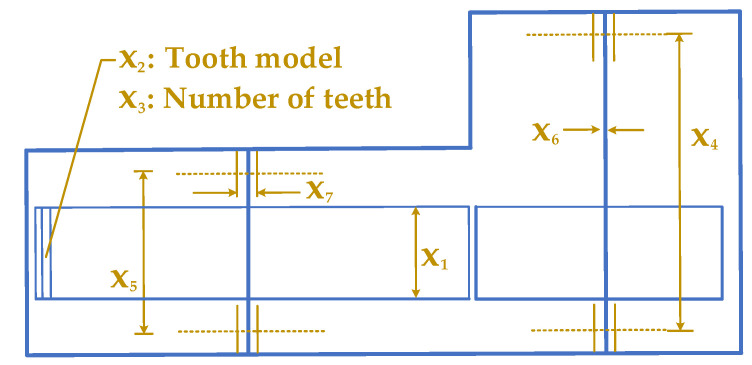
Schematic of SRD.

There are many applications for speed reducers, which is a part of gear boxes within mechanical systems. SRD requires seven design variables, which makes it more difficult [81,82]. Figure 15 presents a diagram of a speed reducer, including its design variables such as the face width ($x_{1}$), the tooth module ($x_{2}$), the number of teeth on the pinion ($x_{3}$), the distance between bearings on the first shaft ($x_{4}$), the distance between bearings on the second shaft ($x_{5}$), the diameter of the first shaft ($x_{6}$), and the diameter of the second shaft ($x_{7}$).

Minimizing the speed reducer’s overall weight whilst meeting eleven limitations would be the goal. Several limitations must be met, consisting of the restrictions on bending stress of gear teeth, surface stress, and transverse deflections of the first and second shafts caused by transmitted force, and stresses in first and second shafts. The mathematical formulation of the SRD problem is presented in the following:
Minimize$f\left(\vec{x}\right)=0.7854x_{1}x_{2}^{2}\left(3.3333x_{3}^{2}+14.9334x_{3}-43.0934\right)-1.508x_{1}\left(x_{6}^{2}+x_{7}^{2}\right)+7.4777\left(x_{6}^{3}+x_{7}^{3}\right)+0.7854(x_{4}x_{6}^{2}+x_{5}x_{7}^{2}),$
Subject to:$\left\{\begin{array}{c} \begin{array}{c} \begin{array}{c} g_{1}\left(\vec{x}\right)=\frac{27}{x_{1}x_{2}^{2}x_{3}}-1\leq 0\\ g_{2}\left(\vec{x}\right)=\frac{397.5}{x_{1}x_{2}^{2}x_{3}^{2}}-1\leq 0\\ g_{3}\left(\vec{x}\right)=\frac{1.93x_{4}^{3}}{x_{2}x_{3}x_{6}^{4}}-1\leq 0 \end{array}\\ g_{4}\left(\vec{x}\right)=\frac{1.93x_{5}^{3}}{x_{2}x_{3}x_{7}^{4}}-1\leq 0\\ g_{5}\left(\vec{x}\right)=\frac{1}{110x_{6}^{3}}\sqrt{{\left(\frac{745x_{4}}{x_{2}x_{3}}\right)}^{2}+16.9\times 10^{6}}-1\leq 0 \end{array}\\ \begin{array}{c} g_{6}\left(\vec{x}\right)=\frac{1}{85x_{7}^{3}}\sqrt{{\left(\frac{745x_{5}}{x_{2}x_{3}}\right)}^{2}+157.5\times 10^{6}}-1\leq 0\\ g_{7}\left(\vec{x}\right)=\frac{x_{2}x_{3}}{40}-1\leq 0\\ g_{8}\left(\vec{x}\right)=\frac{{5x}_{2}}{x_{1}}-1\leq 0 \end{array}\\ \begin{array}{c} g_{9}\left(\vec{x}\right)=\frac{x_{1}}{12x_{2}}-1\leq 0\\ g_{10}\left(\vec{x}\right)=\frac{{1.5x}_{6}+1.9}{x_{4}}-1\leq 0\\ g_{11}\left(\vec{x}\right)=\frac{{1.1x}_{7}+1.9}{x_{5}}-1\leq 0 \end{array} \end{array}\right.$(29)Variable range:$\left\{\begin{array}{c} \begin{array}{c} 2.6\leq x_{1}\leq 3.6\\ 0.7\leq x_{2}\leq 0.8 \end{array}\\ \begin{array}{c} 17\leq x_{3}\leq 28\\ 7.3\leq x_{4}\leq 8.3 \end{array}\\ \begin{array}{c} 7.3\leq x_{5}\leq 8.3\\ 2.9\leq x_{6}\leq 3.9\\ 5\leq x_{7}\leq 5.5 \end{array} \end{array}\right.,$


**Figure 16:**
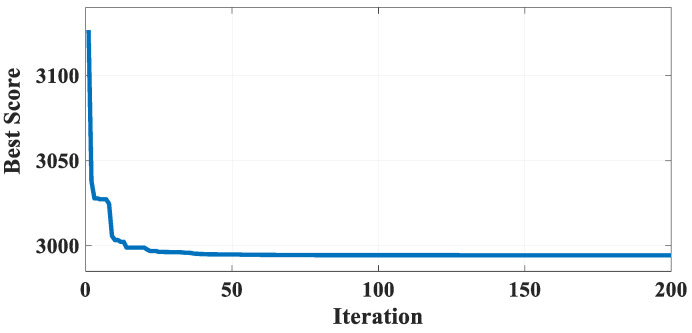
Convergence curve of the GBFIO on the SRD problem.

Table 22 shows the results of GBFIO, GWO, TLBO, DE, and PSO algorithms, which achieved the best solution for SRD variables. The outcomes show that the GBFIO and DE algorithms achieve first rank in finding the best solution for the SRD problem, respectively. As shown in Table 23, the GBFIO algorithm has a low SD equal to 3.02268 × 10^−10^, and it is robust in finding the best solution. Statistical results of the GBFIO algorithm in comparison to other algorithms on the SRD problem are shown in Table 23, which shows that the GBFIO boasts better values for statistical indicators than its competitors. Figure 16 shows the GBFIO convergence curve that achieves optimal values for the SRD problem. A similar penalty function is used for the GBFIO to make accurate comparisons.

## 5. Conclusions

This paper presents and illustrates a novel nature-inspired optimization algorithm inspired by the grizzly bears’ behaviors to increase fat by hunting, fishing, and eating grass, honey, etc. to survive in the winter. An evaluation of 31 benchmark functions was conducted to evaluate the GBFIO’s efficiency in optimization. A comparison is made between the GBFIO’s performance and that of four widely used algorithms—GWO, TLBO, DE, and PSO. A comparative analysis of the achieved ranks, based on both the average of the best solutions and also the best of the best solutions, shows the superior performance of the proposed GBFIO algorithm. For 31 test functions, the average rank based on the best solutions achieved by GBFIO, GWO, TLBO, DE, and PSO is 1.273, 2.659, 3.818, 2.977 and 3.591, respectively. Also, the rank of optimization algorithms based on the best of best solution rank for 31 test functions for GBFIO, GWO, TLBO, DE, and PSO are 1.227, 2.000, 2.864, 2.295, and 3.068, respectively. Furthermore, for the thirty functions of the CEC2017 benchmark function, the rank of optimization algorithms according to the average of the best solution rank for GBFIO, GWO, TLBO, DE, PSO, CPA, TSA, and WOA is 2.233, 3.933, 6.733. 2.933, 4.733, 4, 7.8, and 3.633, respectively. Also, the rank of optimization algorithms based on the best of best solutions rank for GBFIO, GWO, TLBO, DE, PSO, CPA, TSA, and WOA is 2.533, 4.1, 6.767, 2.833, 4.9, 3.533, 7.967, and 3.367, respectively. Hence, the proposed GBFIO is more efficient than other algorithms in solving the abovementioned 61 test functions.

The proposed GBFIO tends to converge to more appropriate quasi-optimal solutions as population sizes increase. Also, by increasing the number of iterations, the proposed GBFIO leads to solutions that are closer to the global optimal. Hence, the suggested algorithm is sensitive to the population size and the iteration number. So, by increasing the population size and the iteration number, the computation time is increased.

According to the outcomes of the simulation, the GBFIO generates effective optimization solutions through a combination of exploration in global search and exploitation in local search. In optimization applications, the GBFIO demonstrated superior performance over other algorithms. Additionally, the GBFIO was implemented to solve four engineering design optimization problems demonstrating that the GBFIO can handle the constrained optimization problems in practice with a low standard deviation, which is indicative of its ability to reach the quasi-optimal solutions.

For further research and future studies, diverse research aspects could be considered, consisting of the design of the multi-objective and binary version of the GBFIO, especially in solving optimization problems in the energy management of smart grids and smart cities; it can also be used for the PMU placement. This paper also proposes using the GBFIO in solving optimization problems in diverse areas of science, suggesting a variety of practical applications.

## Figures and Tables

**Table 1:** Pseudo code of the proposed GBFIO algorithm.

Start the GBFIO algorithm					
Input initial parameters, variables, and constraints					
Generate the initial population randomly within the range of variable values based on Equation (22)					
Compute the objective function					
	For $iteration=1:iteration_{max}$				
		For each population			
Searching Phase			$x_{fish}$, $x_{honey}$, $x_{shell}$, $x_{corpse}$, and $x_{plants}$ are selected as the best top 5 members, receptively.		
			Calculate the new population based on the searching phase by Equations (1)–(11)		
			Compute the objective function		
			If the objective function of the new population < prior objective function		
				Updating the population	
			End if		
Hunt and Care Phases	Hunt Phase		$x_{prey}$ is chosen as the best member of the population		
			Compute the new hunting population based on the hunting phase by Equations (12)–(14)		
	Care Phase		$x_{coyote}$, $x_{cub}^{\left(1\right)}$, and $x_{cub}^{\left(2\right)}$ are chosen as three random members (The best member of these 3 selected members is chosen as coyote, and the other 2 members are bear cubs)		
			Compute the entire new care population based on Equations (15)–(17)		
	Select Phase		Select the new population of the hunt and care phase based on Equation (18).		
			if β≤0.7		
				$x_{bear}^{hunting-care}\left(t+1\right)=x_{bear}^{hunt}\left(t+1\right)$, else	
				$x_{bear}^{hunting-care}\left(t+1\right)=x_{bear}^{care}\left(t+1\right)\;\;\;\;\;if\;\;\;\beta >0.7$	
			End if		
			If the objective function of the new population < prior objective function		
				updating the population	
			End if		
Fishing Phase			Each updated population is a bear that is fishing 25 times each day		
			For i = 1:25		
				Compute the new fishing population by Equation (21)	
				Compute the new objective function	
				If the objective function of the new population < prior objective function	
					Updating the population
				End if	
			End for		
	End for				
	Select the best member of the updated population as a solution				
End the GBFIO algorithm					

**Table 2:** Unimodal benchmark function.

Cost Function	Dimension	Range	$F_{min}$
$CF_{1}(x)=\sum_{l=1}^{k}x_{l}^{2}$	30, 100	[−100, 100]	0
$CF_{2}\left(x\right)=\sum_{l=1}^{k}\left|x_{l}\right|+\prod_{l=1}^{k}\left|x_{l}\right|$	30, 100	[−10, 10]	0
$CF_{3}(x)=\sum_{l=1}^{k}{\left(\sum_{q=1}^{l}x_{l}\right)}^{2}$	30, 100	[−100, 100]	0
$CF_{4}(x)=\max\left\{\left|x_{l}\right|,\;\;1\leq l\leq k\right\}$	30, 100	[−100, 100]	0
$CF_{5}(x)=\sum_{l=1}^{k-1}\left[100{\left(x_{l+1}-x_{l}^{2}\right)}^{2}+{\left(x_{l}-1\right)}^{2}\right]$	30, 100	[−30, 30]	0
$CF_{6}(x)=\sum_{l=1}^{k}{\left(\left|x_{l}+0.5\right|\right)}^{2}$	30, 100	[−100, 100]	0
$CF_{7}(x)=\sum_{l=1}^{k}lx_{l}^{4}+random(0,1)$	30, 100	[−1.28, 1.28]	0

**Table 3:** HDMM test functions.

Cost Function	Dimension	Range	$F_{min}$
$CF_{8}(x)=\sum_{l=1}^{k}-x_{l}\;sin(\sqrt{\left|x_{i}\right|})$	30, 100	[−500, 500]	−418.9829 × Dimension
$CF_{9}(x)=\sum_{l=1}^{k}\left[x_{l}^{2}-10\cos\left(2\pi x_{l}\right)+10\right]$	30, 100	[−5.12, 5.12]	0
$CF_{10}\left(x\right)=-20\exp\left(-0.2\sqrt{\frac{1}{k}\sum_{l=1}^{k}x_{l}^{2}}\right)-\exp\left(\frac{1}{k}\sum_{l=1}^{k}\cos\left(2\pi x_{l}\right)\right)+20+e$	30, 100	[−32, 32]	0
$CF_{11}\left(x\right)=\frac{1}{4000}\sum_{l=1}^{k}x_{l}^{2}-\prod_{l=1}^{k}\cos\left(\frac{x_{l}}{\sqrt{l}}\right)+1$	30, 100	[−600, 600]	0
$CF_{12}\left(x\right)=\frac{\pi }{k}\left\{10\sin^{2}\left(\pi y_{1}\right)+\sum_{l=1}^{k-1}{\left(y_{l}-1\right)}^{2}\left[1+10\sin^{2}\left(\pi y_{l+1}\right)\right]+{\left(y_{k}-1\right)}^{2}\right\}+\sum_{l=1}^{k}u(x_{l},\;10,\;100,\;4)$ $y_{l}=1+\frac{x_{l}+1}{4}$ $u\left(x_{l},\alpha ,p,q\right)=\left\{\begin{array}{c} p{\left(x_{l}-\alpha \right)}^{q},\;\;\;\;\;\;\;\;\;\;x_{l}>\alpha \\ 0,\;\;\;\;\;\;\;\;\;\;-\alpha \leq x_{l}\leq \alpha \\ p{\left(-x_{l}-\alpha \right)}^{q},\;\;\;\;\;\;\;\;\;\;x_{l}<-\alpha \end{array}\right.$	30, 100	[−50, 50]	0
$CF_{13}\left(x\right)=0.1\left\{\sin^{2}\left(3\pi x_{1}\right)+\sum_{l=1}^{k}\begin{array}{c} {\left(x_{l}-1\right)}^{2}\left[1+\sin^{2}\left(3\pi x_{l}+1\right)\right]\\ +{\left(x_{k}-1\right)}^{2}\left[1+\sin^{2}\left(2\pi x_{k}\right)\right] \end{array}\right\}+\sum_{l=1}^{k}u(x_{l},\;5,\;100,\;4)$	30, 100	[−50, 50]	0

**Table 4:** FDMM test functions.

Cost Function	Dimension	Range	$F_{min}$
$CF_{14}\left(x\right)={\left(\frac{1}{500}+\sum_{l=1}^{25}\frac{1}{l+\sum_{k=1}^{2}{\left(x_{k}-a_{kl}\right)}^{6}}\right)}^{-1}$	2	[−65.53, 65.53]	0.998
$a_{kl}=\left[\begin{array}{ccc} \begin{array}{ccc} \begin{array}{c} -32\\ -32 \end{array} & \begin{array}{c} -16\\ -32 \end{array} & \begin{array}{c} 0\\ -32 \end{array} \end{array} & \begin{array}{ccc} \begin{array}{c} 16\\ -32 \end{array} & \begin{array}{c} 32\\ -32 \end{array} & \begin{array}{c} -32\\ -16 \end{array} \end{array} & \begin{array}{ccc} \begin{array}{c} -16\\ -16 \end{array} & \begin{array}{c} 0\\ -16 \end{array} & \begin{array}{cc} \begin{array}{c} 16\\ -16 \end{array} & \begin{array}{ccc} \begin{array}{c} 32\\ -16 \end{array} & \begin{array}{c} -32\\ 0 \end{array} & \begin{array}{ccc} \begin{array}{c} -16\\ 0 \end{array} & \begin{array}{c} 0\\ 0 \end{array} & \begin{array}{ccc} \begin{array}{c} 16\\ 0 \end{array} & \begin{array}{c} 32\\ 0 \end{array} & \begin{array}{ccc} \begin{array}{c} -32\\ 16 \end{array} & \begin{array}{c} -16\\ 16 \end{array} & \begin{array}{ccc} \begin{array}{c} 0\\ 16 \end{array} & \begin{array}{c} 16\\ 16 \end{array} & \begin{array}{ccc} \begin{array}{c} 32\\ 16 \end{array} & \begin{array}{c} -32\\ 32 \end{array} & \begin{array}{c} \begin{array}{ccc} -16 & 0 & \begin{array}{cc} 16 & 32 \end{array} \end{array}\\ \begin{array}{ccc} 32 & 32 & \begin{array}{cc} 32 & 32 \end{array} \end{array} \end{array} \end{array} \end{array} \end{array} \end{array} \end{array} \end{array} \end{array} \end{array} \end{array}\right]$			
$CF_{15}\left(x\right)=\sum_{l=1}^{11}{\left[a_{l}-\frac{x_{1}\left(b_{l}^{2}+b_{l}x_{2}\right)}{b_{l}^{2}+b_{l}x_{3}+x_{4}}\right]}^{2}$	4	[−5, 5]	0.0003
$a_{l}=[\begin{array}{ccc} \begin{array}{ccc} 0.1957 & 0.1947 & 0.1735 \end{array} & \begin{array}{ccc} 0.16 & 0.0844 & 0.0627 \end{array} & \begin{array}{ccc} 0.0456 & 0.0342 & \begin{array}{ccc} 0.0323 & 0.0235 & 0.0246 \end{array} \end{array} \end{array}]$ $b_{l}=[\begin{array}{ccc} \begin{array}{ccc} 4 & 2 & 1 \end{array} & \begin{array}{ccc} \frac{1}{2} & \frac{1}{4} & \frac{1}{6} \end{array} & \begin{array}{ccc} \frac{1}{8} & \frac{1}{10} & \begin{array}{ccc} \frac{1}{12} & \frac{1}{14} & \frac{1}{16} \end{array} \end{array} \end{array}]$			
$CF_{16}\left(x\right)=4x_{1}^{2}-2.1x_{1}^{4}+\frac{1}{3}x_{1}^{6}+x_{1}x_{2}-4x_{2}^{2}+4x_{2}^{4}$	2	[−5, 5]	−1.0316
$CF_{17}\left(x\right)={\left(x_{2}-\frac{5.1}{4\pi^{2}}x_{1}^{2}+\frac{5}{\pi }x_{1}-6\right)}^{2}=10\left(1-\frac{1}{8\pi }\right)cosx_{1}+10$	2	[−5, 5]	0.398
$CF_{18}\left(x\right)=[1+{\left(x_{1}+x_{2}+1\right)}^{2}\left(19-14x_{1}+3x_{1}^{2}-14x_{2}+6x_{1}x_{2}+3x_{2}^{2}\right]*[30+{\left(2x_{1}-3x_{2}\right)}^{2}*(18-32x_{1}+12x_{1}^{2}+48x_{2}-36x_{1}x_{2}+27x_{2}^{2})]$	2	[−5, 5]	3
$CF_{19}\left(x\right)=-\sum_{l=1}^{4}c_{l}exp(-\sum_{k=1}^{3}a_{lk}{\left(x_{k}-P_{lk}\right)}^{2})$	3	[0, 1]	−3.86
$a_{lk}=\left[\begin{array}{cc} \begin{array}{c} 3\\ 0.1 \end{array} & \begin{array}{cc} \begin{array}{c} 10\\ 10 \end{array} & \begin{array}{c} 30\\ 35 \end{array} \end{array}\\ \begin{array}{c} 3\\ 0.1 \end{array} & \begin{array}{cc} \begin{array}{c} 10\\ 10 \end{array} & \begin{array}{c} 30\\ 35 \end{array} \end{array} \end{array}\right]$; $c_{l}=\left[\begin{array}{c} \begin{array}{c} 1\\ 1.2 \end{array}\\ \begin{array}{c} 3\\ 3.2 \end{array} \end{array}\right];\;\;\;\;\;\;P_{lk}=\left[\begin{array}{cc} \begin{array}{c} 0.3689\\ 0.4699 \end{array} & \begin{array}{cc} \begin{array}{c} 0.117\\ 0.4387 \end{array} & \begin{array}{c} 0.2673\\ 0.747 \end{array} \end{array}\\ \begin{array}{c} 0.1091\\ 0.03815 \end{array} & \begin{array}{cc} \begin{array}{c} 0.8732\\ 0.5743 \end{array} & \begin{array}{c} 0.5547\\ 0.8828 \end{array} \end{array} \end{array}\right]$			
$CF_{20}\left(x\right)=-\sum_{l=1}^{4}c_{l}exp(-\sum_{k=1}^{6}a_{lk}{\left(x_{k}-P_{lk}\right)}^{2})$	6	[0, 1]	−3.22
$a_{lk}=\left[\begin{array}{cc} \begin{array}{ccc} 10 & 3 & 17\\ 0.05 & 10 & 17 \end{array} & \begin{array}{ccc} 3.5 & 1.7 & 8\\ 0.1 & 8 & 14 \end{array}\\ \begin{array}{ccc} 3 & 3.5 & 17\\ 17 & 8 & 0.05 \end{array} & \begin{array}{ccc} 10 & 17 & 8\\ 10 & 0.1 & 14 \end{array} \end{array}\right]$; $c_{l}=\left[\begin{array}{c} \begin{array}{c} 1\\ 1.2 \end{array}\\ \begin{array}{c} 3\\ 3.2 \end{array} \end{array}\right];$ $P_{lk}=\left[\begin{array}{cc} \begin{array}{ccc} 0.1312 & 0.16967 & 0.5569\\ 0.2329 & 0.4135 & 0.8307 \end{array} & \begin{array}{ccc} 0.0124 & 0.8283 & 0.5886\\ 0.3736 & 0.1004 & 0.9991 \end{array}\\ \begin{array}{ccc} 0.2348 & 0.1451 & 0.3522\\ 0.4047 & 0.8828 & 0.8732 \end{array} & \begin{array}{ccc} 0.2883 & 0.3047 & 0.6650\\ 0.5743 & 0.1091 & 0.0381 \end{array} \end{array}\right]$			
$CF_{21}\left(x\right)=-\sum_{l=1}^{5}{\left[\left(X-a_{l}\right){\left(X-a_{l}\right)}^{T}+6c_{l}\right]}^{-1}$	4	[0, 10]	−10.1532
$a_{l}=\left[\begin{array}{c} \begin{array}{c} \begin{array}{cc} \begin{array}{ccc} 4 & 4 & 4 \end{array} & 4 \end{array}\\ \begin{array}{cc} \begin{array}{ccc} 1 & 1 & 1 \end{array} & 1 \end{array} \end{array}\\ \begin{array}{c} \begin{array}{cc} \begin{array}{ccc} 8 & 8 & 8 \end{array} & 8 \end{array}\\ \begin{array}{cc} \begin{array}{ccc} 6 & 6 & 6 \end{array} & 6 \end{array} \end{array}\\ \begin{array}{cc} \begin{array}{ccc} 3 & 7 & 3 \end{array} & 7 \end{array} \end{array}\right]$; $c_{l}=\left[\begin{array}{c} \begin{array}{c} 0.1\\ 0.2 \end{array}\\ \begin{array}{c} 0.2\\ 0.4 \end{array}\\ 0.4 \end{array}\right]$			
$CF_{22}\left(x\right)=-\sum_{l=1}^{7}{\left[\left(X-a_{l}\right){\left(X-a_{l}\right)}^{T}+6c_{l}\right]}^{-1}$	4	[0, 10]	−10.4029
$a_{l}=\left[\begin{array}{c} \begin{array}{c} \begin{array}{cc} \begin{array}{ccc} 4 & 4 & 4 \end{array} & 4 \end{array}\\ \begin{array}{cc} \begin{array}{ccc} 1 & 1 & 1 \end{array} & 1 \end{array} \end{array}\\ \begin{array}{c} \begin{array}{cc} \begin{array}{ccc} 8 & 8 & 8 \end{array} & 8 \end{array}\\ \begin{array}{cc} \begin{array}{ccc} 6 & 6 & 6 \end{array} & 6 \end{array} \end{array}\\ \begin{array}{cc} \begin{array}{ccc} \begin{array}{c} 3\\ 2\\ 5 \end{array} & \begin{array}{c} 7\\ 9\\ 3 \end{array} & \begin{array}{c} 3\\ 2\\ 5 \end{array} \end{array} & \begin{array}{c} 7\\ 9\\ 3 \end{array} \end{array} \end{array}\right]$; $c_{l}=\left[\begin{array}{c} \begin{array}{c} 0.1\\ 0.2 \end{array}\\ \begin{array}{c} 0.2\\ 0.4 \end{array}\\ \begin{array}{c} 0.4\\ 0.6\\ 0.3 \end{array} \end{array}\right]$			
$CF_{23}\left(x\right)=-\sum_{l=1}^{10}{\left[\left(X-a_{l}\right){\left(X-a_{l}\right)}^{T}+6c_{l}\right]}^{-1}$	4	[0, 10]	−10.5364
$a_{l}=\left[\begin{array}{cc} \begin{array}{ccc} \begin{array}{c} \begin{array}{c} \begin{array}{c} 4\\ \begin{array}{c} 1\\ 8 \end{array}\\ 6 \end{array}\\ 3 \end{array}\\ 2\\ \begin{array}{c} 5\\ \begin{array}{c} 8\\ 6\\ 7 \end{array} \end{array} \end{array} & \begin{array}{c} \begin{array}{c} \begin{array}{c} 4\\ \begin{array}{c} 1\\ 8 \end{array}\\ 6 \end{array}\\ 7 \end{array}\\ 9\\ \begin{array}{c} 3\\ \begin{array}{c} 1\\ 2\\ 3.6 \end{array} \end{array} \end{array} & \begin{array}{c} \begin{array}{c} \begin{array}{c} 4\\ \begin{array}{c} 1\\ 8 \end{array}\\ 6 \end{array}\\ 3 \end{array}\\ 2\\ \begin{array}{c} 5\\ \begin{array}{c} 8\\ 6\\ 7 \end{array} \end{array} \end{array} \end{array} & \begin{array}{c} \begin{array}{c} \begin{array}{c} 4\\ \begin{array}{c} 1\\ 8 \end{array}\\ 6 \end{array}\\ 7 \end{array}\\ 9\\ \begin{array}{c} 3\\ \begin{array}{c} 1\\ 2\\ 3.6 \end{array} \end{array} \end{array} \end{array}\right]$; $c_{l}=\left[\begin{array}{c} \begin{array}{c} 0.1\\ 0.2 \end{array}\\ \begin{array}{c} 0.2\\ 0.4 \end{array}\\ \begin{array}{c} 0.4\\ 0.6\\ \begin{array}{c} 0.3\\ \begin{array}{c} 0.7\\ 0.5\\ 0.5 \end{array} \end{array} \end{array} \end{array}\right]$			

**Table 5:** The RSB functions.

Cost Function	Dimension	Range	$F_{min}$
${CF}_{24}\left(x\right)=\sin\left(x_{1}\right)e^{\left[{\left(1-cos(x_{2})\right)}^{2}\right]}+\cos\left(x_{2}\right)e^{\left[{\left(1-sin(x_{1})\right)}^{2}\right]}+{\left(x_{1}-x_{2}\right)}^{2}$	2	[−2π, 2π]	−106.764537
${CF}_{25}\left(x\right)=0.5+\frac{\sin^{2}\left(\sqrt{x_{1}^{2}+x_{2}^{2}}\right)}{{\left(1+1\times 10^{-3}(x_{1}^{2}+x_{2}^{2})\right)}^{2}}$	2	[−100, 100]	0.5
${CF}_{26}\left(x\right)=\sum_{i=1}^{k-1}0.5+\frac{\sin^{2}\left(\sqrt{x_{i}^{2}+x_{i+1}^{2}}\right)-0.5}{{\left(1+1\times 10^{-3}(x_{i}^{2}+x_{i+1}^{2})\right)}^{2}}$	20	[−100, 100]	0
${CF}_{27}\left(x\right)=-8|\sin\left(x_{1}\right)\cos\left(x_{2}\right)e^{\left|cos(x_{1}^{2}+x_{2}^{2})/200\right|}|$	2	[−10, 10]	−8.03985
${CF}_{28}\left(x\right)=1\times 10^{-4}{\left(\left|\sin\left(x_{1}\right)\sin\left(x_{2}\right)e^{\left|100-\frac{\sqrt{\left(x_{1}^{2}+x_{2}^{2}\right)}}{\pi }\right|}\right|+1\right)}^{0.1}$	2	[−10, 10]	1.00 × 10^−4^
${CF}_{29}\left(x\right)=1\times 10^{-17}{\left(\left|\sin\left(x_{1}\right)\sin\left(x_{2}\right)e^{\left|100-\frac{\sqrt{\left(x_{1}^{2}+x_{2}^{2}\right)}}{\pi }\right|}\right|\right)}^{0.4}$	40	[−10, 10]	0
${CF}_{30}\left(x\right)=\sum_{i=1}^{k-1}exp(-|\cos\left(x_{1}\right)\cos\left(x_{2}\right)e^{{\left|1-\frac{\sqrt{\left(x_{1}^{2}+x_{2}^{2}\right)}}{\pi }\right|}^{-1}}|)$	50	[−11, 11]	0
${CF}_{31}\left(x\right)=-\left(x2+47\right)\sin\left(\sqrt{\left|x_{2}+\frac{x_{1}}{2}+47\right|}\right)+sin(\sqrt{\left|x_{1}-x_{2}+47\right|})(-x_{1})$	40	[−512, 512]	−955.6087

**Table 6:** Assessment results of UB functions.

		Dimension = 30					Dimension = 100				
		GBFIO	GWO	TLBO	DE	PSO	GBFIO	GWO	TLBO	DE	PSO
$CF_{1}$	Mean	0	6.7809 × 10^−217^	1.3516 × 10^−49^	6.0160 × 10^−18^	0.35710	0	3.5371 × 10^−99^	2.384 × 10^−3^	0.83159	701.973
	SD	0	0	5.6372 × 10^−49^	2.6217 × 10^−18^	0.17647	0	3.6119 × 10^−99^	0.0104	2.5849	135.75
	Best	0	6.77 × 10^−222^	8.52 × 10^−75^	1.77 × 10^−18^	0.11052	0	9.02544 × 10^−101^	2.51064 × 10^−55^	6.86496 × 10^−5^	458.821
	Rank-1	1	2	3	4	5	1	2	3	4	5
	Rank-2	1	2	3	4	5	1	2	3	4	5
$CF_{2}$	Mean	1.1907 × 10^−316^	1.1846 × 10^−107^	1.0374 × 10^−7^	2.9275 × 10^−11^	1.2999	4.57384 × 10^−304^	8.57316 × 10^−55^	2.34113 × 10^−8^	0.00242	33.54858
	SD	0	2.1357 × 10^−107^	4.4653 × 10^−7^	6.7068 × 10^−12^	0.6894	0	1.1311 × 10^−54^	8.0351 × 10^−8^	4.0872 × 10^−4^	4.5074
	Best	4.9766 × 10^−319^	2.3700 × 10^−1104^	3.2400 × 10^−35^	1.2200 × 10^−11^	0.4934	2.26229 × 10^−305^	1.37055 × 10^−55^	7.09498 × 10^−34^	0.00188	25.94422
	Rank-1	1	2	3	4	5	1	2	3	4	5
	Rank-2	1	2	3	4	5	1	2	3	4	5
$CF_{3}$	Mean	0	1.5762 × 10^−28^	0.0048	2766	80.2736	0	49.0044	6250.7	293,331.1	13,741.9
	SD	0	6.6136 × 10^−28^	0.0148	1.0682 × 10^+3^	42.7337	0	96.8952	9837.4	26,324	3896.1
	Best	0	3.1200 × 10^−35^	4.4300 × 10^−12^	1170	29.9414	0	2.03276	12.08	248,065.9	8082.3
	Rank-1	1	2	3	5	4	1	2	3	5	4
	Rank-2	1	2	3	5	4	1	2	3	5	4
$CF_{4}$	Mean	5.4237 × 10^−297^	1.1256 × 10^−30^	3.9335	2.4319	4.5026	1.84971 × 10^−289^	0.04655	10.44494	29.260	18.678
	SD	0	3.0835 × 10^−30^	15.7455	1.6745	1.7914	0	0.0645	22.8253	5.2728	2.529
	Best	7.6100 × 10^−299^	2.1400 × 10^−33^	2.3900 × 10^−20^	0.0113	1.4392	8.62841 × 10^−293^	0.0012	1.82061 × 10^−10^	21.312	13.140
	Rank-1	1	2	3	4	5	1	2	3	5	4
	Rank-2	1	2	3	4	5	1	3	2	5	4
$CF_{5}$	Mean	23.95	25.39	26.565	56.00	104.5487	95.195	95.981	16210.6	819.28	37,611.21
	SD	0.2156	0.5166	0.9366	29.8059	90.8058	0.6437	0.8265	69351	1133.6	16,267
	Best	23.5	24.2	25.5	22	29.1017	94.655	94.741	97.74243	193.38	14,952
	Rank-1	1	2	3	4	5	1	2	4	3	5
	Rank-2	2	3	4	1	5	1	2	3	4	5
$CF_{6}$	Mean	6.8518 × 10^−14^	3.6905 × 10^−6^	2.2210	5.1145 × 10^−19^	0.0250	1.48247	2.97631	17.5878	0.197978	75.23681
	SD	1.3656 × 10^−13^	1.0026 × 10^−6^	0.3736	2.1176 × 10^−19^	0.0161	0.5605	0.8961	0.9210	0.5238	16.0249
	Best	8.8800 × 10^−16^	2.2100 × 10^−6^	1.3400	1.7900 × 10^−19^	0.0028	0.40982	1.74401	15.49978	4.10507 × 10^−6^	45.91405
	Rank-1	2	3	5	1	4	2	3	4	1	5
	Rank-2	2	3	5	1	4	2	3	4	1	5
$CF_{7}$	Mean	3.2825 × 10^−5^	7.3435 × 10^−4^	0.0037	0.0086	0.0183	3.23462 × 10^−5^	7.33905 × 10^−4^	0.00203	0.00953	0.016165
	SD	1.6191 × 10^−5^	2.8128 × 10^−4^	0.0067	0.0014	0.0067	9.7962 × 10^−6^	3.2896 × 10^−4^	0.00105	0.0025	0.0079
	Best	1.1800 × 10^−5^	3.4100 × 10^−4^	3.9900 × 10^−4^	0.0063	0.0043	1.424679 × 10^−5^	2.95192 × 10^−4^	0.00049	0.00464	0.00688
	Rank-1	1	2	3	5	4	1	2	3	4	5
	Rank-2	1	2	3	5	4	1	2	3	4	5

**Table 7:** Assessment results of HDMM functions.

		Dimension = 30					Dimension = 100				
		GBFIO	GWO	TLBO	DE	PSO	GBFIO	GWO	TLBO	DE	PSO
$CF_{8}$	Mean	−9523.5	−6978.5	−4889.5	−8886.4	−6224	−26,531	−19,559	−8781	−12,239.7	−18,962.5
	SD	1754	1012.2	458.044	1480.3	838.1	6045.3	4135	966.5	640.7	2194
	Best	−11,400	−8620	−5770	−11,800	−7889.6	−33,375.9	−22,837.4	−12,274	−13,645.3	−21,910.3
	Rank-1	1	3	5	2	4	1	2	5	4	3
	Rank-2	2	3	5	1	4	1	2	5	4	3
$CF_{9}$	Mean	0	9.2655	158.015	86.585	29.3828	0	12.641	635.0103	697.644	258.951
	SD	0	6.4682	38.4482	13.0317	6.7851	0	8.46656	323.53	29.469	30.445
	Best	0	0	60.3	58.1	18.095	0	0.99759	1.52908	641.77	199.166
	Rank-1	1	2	5	4	3	1	2	4	5	3
	Rank-2	1	1	4	3	2	1	2	3	5	4
$CF_{10}$	Mean	4.4400 × 10^−15^	7.1025 × 10^−15^	8.9155	5.2165 × 10^−10^	3.0216	4.44089 × 10^−15^	2.32703 × 10^−14^	12.8557	3.32459 × 10^−2^	7.9474
	SD	7.8886 × 10^−31^	1.5372 × 10^−15^	8.1385	1.7844 × 10^−10^	0.7375	0	4.06627 × 10^−15^	8.85245	8.03283 × 10^−2^	0.65451
	Best	4.4400 × 10^−15^	4.4400 × 10^−15^	4.4400 × 10^−15^	2.9400 × 10^−10^	1.5185	4.44089 × 10^−15^	1.50990 × 10^−14^	7.99361 × 10^−15^	9.87967 × 10^−4^	6.72938
	Rank-1	1	2	5	3	4	1	2	5	3	4
	Rank-2	1	1	1	2	3	1	3	2	4	5
$CF_{11}$	Mean	0	0.0055	0.0197	0	0.2906	0	1.08646 × 10^−3^	1.84587 × 10^−6^	2.87534 × 10^−3^	8.41713
	SD	0	0.0092	0.0793	0	0.1456	0	3.26004 × 10^−3^	8.04594 × 10^−6^	6.18704 × 10^−3^	1.44086
	Best	0	0	0	0	0.0617	0	0	0	3.57892 × 10^−5^	6.13197
	Rank-1	1	3	2	1	4	1	3	2	4	5
	Rank-2	1	1	1	1	2	1	1	1	2	3
$CF_{12}$	Mean	5.5608 × 10^−16^	0.0013	0.1460	5.8625 × 10^−19^	1.4325	6.52623 × 10^−3^	5.37489 × 10^−2^	2.02809 × 10^+7^	1.51014	12.39863
	SD	5.4263 × 10^−16^	0.0027	0.0559	4.7541 × 10^−19^	1.1414	2.77643 × 10^−3^	1.58948 × 10^−2^	8.83913 × 10^+7^	5.23148	4.24249
	Best	4.9600 × 10^−17^	1.1400 × 10^−7^	0.0785	1.1700 × 10^−19^	0.3464	2.19108 × 10^−3^	3.30271 × 10^−2^	5.10567 × 10^−1^	2.53465 × 10^−5^	6.641798
	Rank-1	2	3	4	1	5	1	2	5	3	4
	Rank-2	2	3	4	1	5	2	3	4	1	5
$CF_{13}$	Mean	3.3100 × 10^−5^	0.0252	1.5150	0.0530	3.9508	2.98239	2.93852	12.63706	396.468	240.727
	SD	1.4428 × 10^−4^	0.0481	0.2505	0.2265	2.9004	0.5132	0.57558	7.56417	1228.85	131.559
	Best	9.9300 × 10^−15^	2.8800 × 10^−6^	1.0700	2.4600 × 10^−18^	0.6260	2.0708	1.87816	9.77515	0.315757	156.342
	Rank-1	1	2	4	3	5	2	1	3	5	4
	Rank-2	2	3	5	1	4	3	2	4	1	5

**Table 8:** Assessment results of FDMM functions.

		GBFIO	GWO	TLBO	DE	PSO
$CF_{14}$	Mean	0.998003838	0.998003838	0.99800467	0.998003838	1.14710789
	SD	0	6.1799 × 10^−12^	2.7057 × 10^−6^	0	0.354938656
	Best	0.998003838	0.998003838	0.998003838	0.998003838	0.998003838
	Rank-1	1	2	3	1	4
	Rank-2	1	1	1	1	1
$CF_{15}$	Mean	3.07 × 10^−4^	0.0033	5.7905 × 10^−4^	5.8005 × 10^−4^	4.4484 × 10^−4^
	SD	0	0.0072	2.7999 × 10^−4^	1.3945 × 10^−4^	3.2697 × 10^−4^
	Best	3.07 × 10^−4^	3.07 × 10^−4^	3.09 × 10^−4^	3.07 × 10^−4^	3.0749 × 10^−4^
	Rank-1	1	5	3	4	2
	Rank-2	1	1	3	1	2
$CF_{16}$	Mean	−1.0316	−1.0316	−1.0316	−1.0316	−1.0316
	SD	2.1642 × 10^−16^	7.5654 × 10^−10^	7.1017 × 10^−5^	2.2204 × 10^−16^	2.1642 × 10^−16^
	Best	−1.0316	−1.0316	−1.0316	−1.0316	−1.0316
	Rank-1	1	3	4	2	1
	Rank-2	1	1	1	1	1
$CF_{17}$	Mean	0. 39788736	0.39788738	0.39788738	0.39788736	0.39788736
	SD	0	2.6038 × 10^−8^	4.3017 × 10^−8^	0	0
	Best	0.39788736	0.39788736	0.39788736	0.39788736	0.39788736
	Rank-1	1	2	3	1	1
	Rank-2	1	1	1	1	1
$CF_{18}$	Mean	2.99999999999992	3.00000005812866	3.00017061441758	2.99999999999992	2.99999999999992
	SD	0	9.8523 × 10^−8^	4.4211 × 10^−4^	9.0468 × 10^−16^	2.2204 × 10^−16^
	Best	2.99999999999992	3.00000000025439	2.99999999999995	2.99999999999992	2.99999999999992
	Rank-1	1	4	5	3	2
	Rank-2	1	3	2	1	1
$CF_{19}$	Mean	−3.862782	−3.862779	−3.862460	−3.862782	−3.862782
	SD	2.2204 × 10^−15^	6.0993 × 10^−6^	0.0012	2.2204 × 10^−15^	2.2004 × 10^−15^
	Best	−3.862782	−3.862782	−3.862782	−3.862782	−3.862782
	Rank-1	2	3	4	2	1
	Rank-2	1	1	1	1	1
$CF_{20}$	Mean	−3.207927	−3.196545	−3.168953	−3.202468	−3.204332
	SD	0.0087	0.022283	0.034273	0.006574	0.008985
	Best	−3.222190	−3.222189	−3.207691	−3.222190	−3.222190
	Rank-1	1	4	5	3	2
	Rank-2	1	2	3	1	1
$CF_{21}$	Mean	−10.153200	−9.053145	−8.791638	−9.895080	−8.272518
	SD	2.8087 × 10^−15^	2.2271	1.4955	1.1002	3.2574
	Best	−10.153200	−10.153193	−10.121451	−10.153200	−10.153200
	Rank-1	1	3	4	2	5
	Rank-2	1	2	3	1	1
$CF_{22}$	Mean	−10.402915	−10.139136	−9.467246	−10.402915	−10.020248
	SD	1.7764 × 10^−15^	1.1495	1.2249	1.7764 × 10^−15^	1.6680
	Best	−10.402915	−10.402912	−10.400771	−10.402915	−10.402915
	Rank-1	1	2	4	1	3
	Rank-2	1	2	3	1	1
$CF_{23}$	Mean	−10.536443	−10.536363	−9.500209	−9.945075	−10.536443
	SD	1.1916 × 10^−15^	7.7539 × 10^−5^	1.3665	1.7827	1.1235 × 10^−15^
	Best	−10.536443	−10.536440	−10.535909	−10.536443	−10.536443
	Rank-1	2	3	5	4	1
	Rank-2	1	2	3	1	1

**Table 9:** Assessment results of RSB functions.

		GBFIO	GWO	TLBO	DE	PSO
$CF_{24}$	Mean	−106.764537	−106.764532	−106.75358	−106.764537	−106.764537
	SD	1.5723 × 10^−9^	7.3496 × 10^−6^	0.032729	2.7335 × 10^−14^	2.9296 × 10^−14^
	Best	−106.764537	−106.764537	−106.764537	−106.764537	−106.764537
	Rank-1	3	4	5	1	2
	Rank-2	1	1	1	1	1
$CF_{25}$	Mean	0.5000	0.5000	0.5000	0.5000	0.5000
	SD	1.6784 × 10^−14^	1.0430 × 10^−11^	2.5923 × 10^−12^	0	0
	Best	0.5000	0.5000	0.5000	0.5000	0.5000
	Rank-1	2	4	3	1	1
	Rank-2	1	1	1	1	1
$CF_{26}$	Mean	3.993739	4.242761	6.757190	5.419060	4.747401
	SD	0.850113	1.13408372494118	0.558152	0.270471	0.761412
	Best	1.805434	2.40637	5.698089	4.933010	3.215879
	Rank-1	1	2	5	4	3
	Rank-2	1	2	5	4	3
$CF_{27}$	Mean	−8.03985	−8.03922	−8.03937	−8.03983	−8.03898
	SD	8.76466 × 10^−6^	3.89416 × 10^−4^	4.94818 × 10^−4^	8.60975 × 10^−5^	1.40674 × 10^−3^
	Best	−8.03985	−8.03985	−8.03985	−8.03985	−8.03945
	Rank-1	1	4	3	2	5
	Rank-2	1	1	1	1	1
$CF_{28}$	Mean	0.03464	0.37738	0.31450	0.00037	0.00055
	SD	0.05045	0.09128	0.06964	0.00047	0.00055
	Best	1.00 × 10^−4^	0.234437	0.173517	1.00 × 10^−4^	1.00 × 10^−4^
	Rank-1	3	5	4	1	2
	Rank-2	1	3	2	1	1
$CF_{29}$	Mean	0.01795	4.14519	7.85220	4.50203	4.23656
	SD	0.01091	4.21639	0.31422	0.42907	0.80989
	Best	0.0051	0.70668	6.78426	3.55829	2.97515
	Rank-1	1	2	5	4	3
	Rank-2	1	2	5	4	3
$CF_{30}$	Mean	0.117687	0.407370	1.056183	0.006566	0.386806
	SD	0.086418	0.160198	1.186894	0.006526	0.184927
	Best	6.492597 × 10^−5^	0.165007	0.297562	1.677507 × 10^−5^	0.110005
	Rank-1	2	4	5	1	3
	Rank-2	2	4	5	1	3
$CF_{31}$	Mean	−955.608723	−953.013547	−955.089361	−955.089692	−884.819663
	SD	1.68625 × 10^−13^	4.49498	2.26251	2.262402	83.64259
	Best	−955.608723	−955.608723	−955.608723	−955.608723	−955.608723
	Rank-1	1	4	3	2	5
	Rank-2	1	1	1	1	1

**Table 10:** *p*-values acquired from the Wilcoxon sum rank test.

Function	Dimension = 100							
	$CF_{1}$	$CF_{2}$	$CF_{3}$	$CF_{4}$	$CF_{5}$	$CF_{6}$	$CF_{7}$	$CF_{8}$
GBFIO V.S. GWO	8.0065 × 10^−9^	6.7860 × 10^−8^	7.9919 × 10^−9^	6.7956 × 10^−8^	9.0289 × 10^−4^	2.3531 × 10^−6^	6.7860 × 10^−8^	9.7106 × 10^−6^
GBFIO V.S. TLBO	8.0065 × 10^−9^	6.7860 × 10^−8^	8.0065 × 10^−9^	6.7956 × 10^−8^	6.1179 × 10^−8^	6.7765 × 10^−8^	6.7860 × 10^−8^	9.1222 × 10^−8^
GBFIO V.S. DE	7.9919 × 10^−9^	6.7860 × 10^−8^	7.9626 × 10^−9^	6.7860 × 10^−8^	6.4949 × 10^−8^	1.8030 × 10^−6^	6.7860 × 10^−8^	1.5750 × 10^−5^
GBFIO V.S. PSO	8.0065 × 10^−9^	6.7860 × 10^−8^	8.0065 × 10^−9^	6.7956 × 10^−8^	6.4949 × 10^−8^	6.7956 × 10^−8^	6.7860 × 10^−8^	1.5983 × 10^−5^
**Function**	**Dimension = 100**							
	$CF_{9}$	$CF_{10}$	$CF_{11}$	$CF_{12}$	$CF_{13}$	$CF_{14}$	$CF_{15}$	$CF_{16}$
GBFIO V.S. GWO	7.9480 × 10^−9^	3.8352 × 10^−9^	0.1626	6.7765 × 10^−8^	0.8711	N/A	0.0804	N/A
GBFIO V.S. TLBO	8.0065 × 10^−9^	7.6327 × 10^−9^	0.1626	6.7860 × 10^−8^	6.7288 × 10^−8^	N/A	7.9919 × 10^−9^	N/A
GBFIO V.S. DE	7.9919 × 10^−9^	7.9626 × 10^−9^	8.0065 × 10^−9^	2.9223 × 10^−5^	0.0017	N/A	2.9868 × 10^−8^	N/A
GBFIO V.S. PSO	8.0065 × 10^−9^	8.0065 × 10^−9^	8.0065 × 10^−9^	6.7956 × 10^−8^	6.7956 × 10^−8^	1.5427 × 10^−9^	1.5427 × 10^−9^	4.6827 × 10^−10^
**Function**	$CF_{17}$	$CF_{18}$	$CF_{19}$	$CF_{20}$	$CF_{21}$	$CF_{22}$	$CF_{23}$	$CF_{24}$
GBFIO V.S. GWO	N/A	N/A	N/A	0.0393	0.0400	0.3421	N/A	N/A
GBFIO V.S. TLBO	N/A	N/A	N/A	6.9726 × 10^−5^	7.9480 × 10^−9^	1.0381 × 10^−7^	1.0968 × 10^−6^	N/A
GBFIO V.S. DE	N/A	N/A	N/A	0.0236	0.1626	N/A	0.1626	N/A
GBFIO V.S. PSO	4.6827 × 10^−10^	N/A	4.6827 × 10^−10^	0.3481	2.5780 × 10^−9^	3.0335 × 10^−8^	4.6827 × 10^−10^	4.6827 × 10^−10^
**Function**	$CF_{25}$	$CF_{26}$	$CF_{27}$	$CF_{28}$	$CF_{29}$	$CF_{30}$	$CF_{31}$	
GBFIO V.S. GWO	N/A	0.3792	N/A	5.8435 × 10^−8^	6.7860 × 10^−8^	4.2490 × 10^−6^	0.0195	
GBFIO V.S. TLBO	N/A	6.7765 × 10^−8^	N/A	5.8519 × 10^−8^	6.7765 × 10^−8^	1.0602 × 10^−7^	0.3421	
GBFIO V.S. DE	N/A	4.5110 × 10^−7^	N/A	5.5870 × 10^−4^	6.7669 × 10^−8^	9.1601 × 10^−8^	0.3421	
GBFIO V.S. PSO	N/A	0.0133	3.1997 × 10^−9^	0.0114	6.7956 × 10^−8^	1.5961 × 10^−4^	2.1875 × 10^−8^	

**Table 11:** Results of the GBFIO algorithm sensitivity analysis with respect to population sizes.

		Number of Population				
		20	30	40	50	60
$CF_{1}$	Mean	0	0	0	0	0
	SD	0	0	0	0	0
	Best	0	0	0	0	0
$CF_{2}$	Mean	9.488376 × 10^−308^	8.961751 × 10^−312^	4.425393 × 10^−312^	2.964866 × 10^−312^	4.131339 × 10^−314^
	SD	0	0	0	0	0
	Best	4.970226 × 10^−313^	1.223130 × 10^−313^	2.260542 × 10^−314^	5.041268 × 10^−315^	2.445212 × 10^−316^
$CF_{3}$	Mean	0	0	0	0	0
	SD	0	0	0	0	0
	Best	0	0	0	0	0
$CF_{4}$	Mean	3.155977 × 10^−290^	7.658761 × 10^−291^	2.641380 × 10^−293^	1.301568 × 10^−293^	1.090979 × 10^−293^
	SD	0	0	0	0	0
	Best	5.607299 × 10^−296^	4.390067 × 10^−295^	4.779610 × 10^−296^	1.083217 × 10^−296^	5.658902 × 10^−297^
$CF_{5}$	Mean	24.847301	24.592571	24.504815	24.359246	24.409804
	SD	0.405346	0.304791	0.297845	0.328575	0.286390
	Best	24.277476	23.940380	24.045424	23.909135	23.919656
$CF_{6}$	Mean	2.174950 × 10^−9^	1.427397 × 10^−10^	4.364226 × 10^−11^	1.444414 × 10^−11^	8.657999 × 10^−12^
	SD	2.416979 × 10^−9^	1.508869 × 10^−10^	3.702102 × 10^−11^	1.369896 × 10^−11^	8.307039 × 10^−12^
	Best	6.53199 × 10^−11^	6.958819 × 10^−12^	6.127176 × 10^−12^	9.838662 × 10^−13^	8.529919 × 10^−13^
$CF_{7}$	Mean	7.968518 × 10^−5^	7.081454 × 10^−5^	5.588143 × 10^−5^	6.343727 × 10^−5^	4.857298 × 10^−5^
	SD	4.791609 × 10^−5^	3.430162 × 10^−5^	2.361767 × 10^−5^	2.313969 × 10^−5^	2.589811 × 10^−5^
	Best	1.383589 × 10^−5^	1.308217 × 10^−5^	2.123137 × 10^−5^	2.875731 × 10^−5^	1.143129 × 10^−5^
$CF_{8}$	Mean	−7650.140234	−8264.838722	−8280.885116	−8209.417095	−8964.229479
	SD	2173.569713	1805.621009	1952.301928	2257.507354	2127.146066
	Best	−10,376.851123	−10,428.494237	−11,143.987174	−11,067.745203	−11,403.276763
$CF_{9}$	Mean	8.754598	6.765642	5.662471	0	0
	SD	23.202940	17.050401	17.419375	0	0
	Best	0	0	0	0	0
$CF_{10}$	Mean	4.440892 × 10^−15^	4.440892 × 10^−15^	4.440892 × 10^−15^	4.440892 × 10^−15^	4.440892 × 10^−15^
	SD	0	0	0	0	0
	Best	4.440892 × 10^−15^	4.440892 × 10^−15^	4.440892 × 10^−15^	4.440892 × 10^−15^	4.440892 × 10^−15^
$CF_{11}$	Mean	0	0	0	0	0
	SD	0	0	0	0	0
	Best	0	0	0	0	0
$CF_{12}$	Mean	6.271662 × 10^−11^	8.523194 × 10^−12^	2.468391 × 10^−12^	6.364377 × 10^−13^	1.200745 × 10^−13^
	SD	7.522505 × 10^−11^	1.295646 × 10^−11^	5.984179 × 10^−12^	8.595888 × 10^−13^	1.360803 × 10^−13^
	Best	8.262464 × 10^−13^	2.042665 × 10^−13^	6.735877 × 10^−14^	3.604206 × 10^−14^	5.514638 × 10^−15^
$CF_{13}$	Mean	0.197081	0.112027	0.106591	0.077995	0.038914
	SD	0.132252	0.111567	0.098950	0.075663	0.065934
	Best	6.583484 × 10^−10^	2.716825 × 10^−10^	2.515858 × 10^−11^	1.976762 × 10^−11^	1.399126 × 10^−12^
$CF_{14}$	Mean	0.998003838	0.998003838	0.998003838	0.998003838	0.998003838
	SD	9.930137 × 10^−17^	0	7.021667 × 10^−17^	0	0
	Best	0.998003838	0.998003838	0.998003838	0.998003838	0.998003838
$CF_{15}$	Mean	3.461836 × 10^−4^	3.152664 × 10^−4^	3.113838 × 10^−4^	3.168474 × 10^−4^	3.084805 × 10^−4^
	SD	7.297051 × 10^−5^	1.975967 × 10^−5^	1.318464 × 10^−5^	3.184629 × 10^−5^	3.802653 × 10^−6^
	Best	3.074859 × 10^−4^	3.074859 × 10^−4^	3.074859 × 10^−4^	3.074859 × 10^−4^	3.074859 × 10^−4^
$CF_{16}$	Mean	−1.031628	−1.031628	−1.031628	−1.031628	−1.031628
	SD	3.729863 × 10^−9^	1.876679 × 10^−9^	2.220446 × 10^−16^	2.220446 × 10^−16^	2.220446 × 10^−16^
	Best	−1.031628	−1.031628	−1.031628	−1.031628	−1.031628
$CF_{17}$	Mean	0.39788736	0.39788736	0.39788736	0.39788736	0.39788736
	SD	0	0	0	0	0
	Best	0.39788736	0.39788736	0.39788736	0.39788736	0.39788736
$CF_{18}$	Mean	2.999999999999922	2.999999999999922	2.999999999999922	2.999999999999922	2.99999999999992
	SD	8.992121 × 10^−16^	4.965068 × 10^−16^	5.063396 × 10^−16^	4.550560 × 10^−16^	3.140185 × 10^−16^
	Best	2.999999999999921	2.999999999999922	2.999999999999921	2.999999999999921	2.999999999999921
$CF_{19}$	Mean	−3.862782	−3.862782	−3.862782	−3.862782	−3.862782
	SD	2.220446 × 10^−15^	2.220446 × 10^−15^	2.220446 × 10^−15^	2.220446 × 10^−15^	2.220446 × 10^−15^
	Best	−3.862782	−3.862782	−3.862782	−3.862782	−3.862782
$CF_{20}$	Mean	−3.204730	−3.203979	−3.207953	−3.204986	−3.204392
	SD	0.018867	0.018451	0.010447	0.008771	0.018489
	Best	−3.222190	−3.222190	−3.222190	−3.222190	−3.222190
$CF_{21}$	Mean	−9.286335	−9.583326	−9.894665	−10.132708	−10.141487
	SD	1.789232	1.518792	1.110277	0.030605	0.035946
	Best	−10.1531996790582	−10.1531996790582	−10.1531996790582	−10.1531996790582	−10.1531996790582
$CF_{22}$	Mean	−9.809420	−10.381395	−10.384025	−10.401542	−10.402913
	SD	1.592699	0.057163	0.051033	0.005985	1.152257 × 10^−5^
	Best	−10.402915	−10.402915	−10.402915	−10.402915	−10.402915
$CF_{23}$	Mean	−9.474190	−10.207301	−10.264706	−10.529081	−10.536443
	SD	2.180221	1.408249	1.178794	0.030607	8.881784 × 10^−16^
	Best	−10.536443	−10.536443	−10.536443	−10.536443	−10.536443
$CF_{24}$	Mean	−106.764537	−106.764537	−106.764537	−106.764537	−106.764537
	SD	2.313359 × 10^−14^	2.377931 × 10^−14^	3.759839 × 10^−14^	2.561898 × 10^−14^	3.552714 × 10^−14^
	Best	−106.764537	−106.764537	−106.764537	−106.764537	−106.764537
$CF_{25}$	Mean	0.500000000000311	0.500000000000209	0.500000000000182	0.500000000000043	0.500000000000040
	SD	3.939481 × 10^−13^	3.403677 × 10^−13^	4.809765 × 10^−13^	6.064783 × 10^−14^	4.258445 × 10^−14^
	Best	0.500000000000001	0.500000000000000	0.500000000000000	0.500000000000000	0.500000000000000
$CF_{26}$	Mean	4.709092	5.105572	4.897150	4.662565	4.565587
	SD	0.700081	0.545440	0.547182	0.712527	0.738187
	Best	3.150080	3.901535	3.844543	3.337249	3.091730
$CF_{27}$	Mean	−8.039597	−8.039755	−8.039829	−8.039806	−8.039810
	SD	0.000227	0.000158	4.728803 × 10^−5^	9.869128 × 10^−5^	7.925843 × 10^−5^
	Best	−8.03985	−8.03985	−8.03985	−8.03985	−8.03985
$CF_{28}$	Mean	0.121397	0.053492	0.053453	0.052545	0.055992
	SD	0.086411	0.063682	0.064224	0.070996	0.056458
	Best	0.0001	0.0001	0.0001	0.0001	0.0001
$CF_{29}$	Mean	0.165859	0.124924	0.065325	0.072015	0.041180
	SD	0.198043	0.156524	0.040186	0.117556	0.022127
	Best	0.018643	0.012714	0.013105	0.005866	0.005531
$CF_{30}$	Mean	0.300939	0.271066	0.182162	0.231211	0.196458
	SD	0.119096	0.108416	0.092769	0.111651	0.093854
	Best	0.109866	0.115039	0.062385	0.007016	0.004542
$CF_{31}$	Mean	−955.608723	−955.608723	−955.608723	−955.608723	−955.608723
	SD	1.368971 × 10^−13^	1.219156 × 10^−13^	1.296229 × 10^−13^	1.368971 × 10^−13^	1.296229 × 10^−13^
	Best	−955.608723	−955.608723	−955.608723	−955.608723	−955.608723

**Table 12:** Results of the GBFIO algorithm sensitivity analysis with respect to the maximum number of iterations.

		Number of Maximum Iterations				
		50	100	200	500	1000
$CF_{1}$	Mean	7.236219 × 10^−60^	4.888781 × 10^−122^	2.723329 × 10^−245^	0	0
	SD	1.329818 × 10^−59^	5.664636 × 10^−122^	0	0	0
	Best	8.282774 × 10^−62^	1.502527 × 10^−123^	3.714608 × 10^−248^	0	0
$CF_{2}$	Mean	8.077162 × 10^−31^	3.011036 × 10^−62^	2.954380 × 10^−125^	1.095445 × 10^−314^	0
	SD	5.150460 × 10^−31^	3.159078 × 10^−62^	6.131570 × 10^−125^	0	0
	Best	1.799495 × 10^−31^	4.422890 × 10^−63^	1.000961 × 10^−126^	5.244914 × 10^−317^	0
$CF_{3}$	Mean	2.663880 × 10^−56^	7.335968 × 10^−115^	2.753337 × 10^−230^	0	0
	SD	3.661737 × 10^−56^	1.226888 × 10^−114^	0	0	0
	Best	1.716129 × 10^−58^	1.095871 × 10^−117^	2.055096 × 10^−236^	0	0
$CF_{4}$	Mean	5.417876 × 10^−29^	9.849815 × 10^−59^	1.081251 × 10^−117^	9.603995 × 10^−295^	1.000000 × 10^−323^
	SD	6.091838 × 10^−29^	1.687004 × 10^−58^	9.333747 × 10^−118^	0	0
	Best	3.866891 × 10^−30^	1.421012 × 10^−61^	6.562393 × 10^−119^	1.265138 × 10^−297^	1.000000 × 10^−323^
$CF_{5}$	Mean	26.813010	26.269416	25.537543	24.219361	23.006166
	SD	0.229245	0.166345	0.2252578	0.258855	0.393699
	Best	26.396138	25.898832	25.081468	23.790486	22.436206
$CF_{6}$	Mean	0.174425	0.009292	2.603807 × 10^−5^	1.459834 × 10^−12^	8.525488 × 10^−25^
	SD	0.095731	0.004469	1.775197 × 10^−5^	1.347648 × 10^−12^	1.707910 × 10^−24^
	Best	0.053348	0.003414	8.222699 × 10^−6^	1.073916 × 10^−13^	5.523253 × 10^−27^
$CF_{7}$	Mean	4.157443 × 10^−4^	2.363886 × 10^−4^	1.135765 × 10^−4^	4.609243 × 10^−5^	2.655697 × 10^−5^
	SD	2.696449 × 10^−4^	1.036415 × 10^−4^	4.512791 × 10^−5^	1.712571 × 10^−5^	1.169044 × 10^−5^
	Best	8.646435 × 10^−5^	2.857222 × 10^−5^	2.400965 × 10^−5^	2.204244 × 10^−5^	5.296813 × 10^−6^
$CF_{8}$	Mean	−5522.811083	−6649.882116	−7979.289336	−8349.210891	−9603.189247
	SD	1618.143281	2108.537271	2282.800575	2202.544287	1486.878032
	Best	−10,333.978992	−10,281.485338	−11,297.097530	−11,403.322725	−11,621.979941
$CF_{9}$	Mean	12.276021	4.377582	4.236812	0	0
	SD	36.829146	19.081437	18.467836	0	0
	Best	0	0	0	0	0
$CF_{10}$	Mean	5.684342 × 10^−15^	4.440892 × 10^−15^	4.440892 × 10^−15^	4.440892 × 10^−15^	4.440892 × 10^−15^
	SD	1.694536 × 10^−15^	0	0	0	0
	Best	4.440892 × 10^−15^	4.440892 × 10^−15^	4.440892 × 10^−15^	4.440892 × 10^−15^	4.440892 × 10^−15^
$CF_{11}$	Mean	0	0	0	0	0
	SD	0	0	0	0	0
	Best	0	0	0	0	0
$CF_{12}$	Mean	0.006145	2.496981 × 10^−4^	6.758787 × 10^−7^	4.177336 × 10^−14^	1.819623 × 10^−26^
	SD	0.003086	1.323707 × 10^−4^	3.414239 × 10^−7^	9.785659 × 10^−14^	4.362844 × 10^−26^
	Best	0.001692	8.998801 × 10^−5^	2.097865 × 10^−7^	1.334239 × 10^−15^	2.218079 × 10^−28^
$CF_{13}$	Mean	0.237284	0.093105	0.027542	0.034877	0.011916
	SD	0.085985	0.101294	0.045562	0.063506	0.040562
	Best	0.085086	0.009021	2.653115 × 10^−5^	3.509161 × 10^−13^	2.273020 × 10^−25^
$CF_{14}$	Mean	0.998003838	0.998003838	0.998003838	0.998003838	0.998003838
	SD	2.106500 × 10^−16^	1.216188 × 10^−16^	0	0	0
	Best	0.998003838	0.998003838	0.998003838	0.998003838	0.998003838
$CF_{15}$	Mean	3.749509 × 10^−4^	3.111576 × 10^−4^	3.076168 × 10^−4^	3.075054 × 10^−4^	3.074910 × 10^−4^
	SD	2.001892 × 10^−4^	1.428813 × 10^−5^	4.882946 × 10^−7^	5.363649 × 10^−8^	2.195724 × 10^−8^
	Best	3.074867 × 10^−4^	3.074861 × 10^−4^	3.074860 × 10^−4^	3.074860 × 10^−4^	3.074860 × 10^−4^
$CF_{16}$	Mean	−1.031628	−1.031628	−1.031628	−1.031628	−1.031628
	SD	1.140715 × 10^−7^	1.453566 × 10^−9^	7.645066 × 10^−11^	2.220446 × 10^−16^	2.220446 × 10^−16^
	Best	−1.031628	−1.031628	−1.031628	−1.031628	−1.031628
$CF_{17}$	Mean	0.39788736	0.39788736	0.39788736	0.39788736	0.39788736
	SD	0	0	0	0	0
	Best	0.39788736	0.39788736	0.39788736	0.39788736	0.39788736
$CF_{18}$	Mean	2.999999999999930	2.999999999999923	2.999999999999922	2.999999999999922	2.999999999999922
	SD	5.504736 × 10^−15^	1.731378 × 10^−15^	8.770059 × 10^−16^	4.094300 × 10^−16^	5.063396 × 10^−16^
	Best	2.999999999999923	2.999999999999922	2.999999999999921	2.999999999999921	2.999999999999920
$CF_{19}$	Mean	−3.862782	−3.862782	−3.862782	−3.862782	−3.862782
	SD	2.180112 × 10^−15^	1.756821 × 10^−15^	2.220446 × 10^−15^	2.220446 × 10^−15^	2.220446 × 10^−15^
	Best	−3.862782	−3.862782	−3.862782	−3.862782	−3.862782
$CF_{20}$	Mean	−3.202283	−3.203573	−3.205763	−3.205044	−3.203534
	SD	6.689631 × 10^−3^	7.820802 × 10^−3^	9.484115 × 10^−3^	8.443659 × 10^−3^	7.729254 × 10^−3^
	Best	−3.222190	−3.222190	−3.222190	−3.222190	−3.222190
$CF_{21}$	Mean	−8.777263	−9.876641	−10.093695	−10.147108	−10.152762
	SD	1.800773	0.654818	0.232526	2.524644 × 10^−2^	1.909355 × 10^−3^
	Best	−10.1531996790505	−10.1531996790582	−10.1531996790582	−10.1531996790582	−10.1531996790582
$CF_{22}$	Mean	−10.015114	−10.054852	−10.374473	−10.402915	−10.402915
	SD	1.025274	1.176498	0.123977	1.863059 × 10^−15^	1.685200 × 10^−15^
	Best	−10.402915	−10.402915	−10.402915	−10.402915	−10.402915
$CF_{23}$	Mean	−9.776116	−10.162331	−10.536443	−10.536443	−10.53644
	SD	1.838704	1.207934	3.445696 × 10^−10^	1.432145 × 10^−15^	1.191616 × 10^−15^
	Best	−10.536443	−10.536443	−10.536443	−10.536443	−10.536443
$CF_{24}$	Mean	−106.764536	−106.764536	−106.764537	−106.764537	−106.764537
	SD	2.747015 × 10^−6^	2.350338 × 10^−6^	1.903775 × 10^−7^	3.177644 × 10^−14^	2.929643 × 10^−14^
	Best	−106.764537	−106.764537	−106.764537	−106.764537	−106.764537
$CF_{25}$	Mean	0.500000000004132	0.500000000000730	0.500000000000137	0.500000000000084	0.500000000000009
	SD	6.984784 × 10^−12^	9.168767 × 10^−13^	2.589352 × 10^−13^	1.275293 × 10^−13^	1.343753 × 10^−14^
	Best	0.500000000000003	0.500000000000002	0.500000000000001	0.500000000000000	0.500000000000000
$CF_{26}$	Mean	5.910513	5.469769	4.974104	4.642032	3.924982
	SD	0.602621	0.601245	0.555017	0.446079	0.536122
	Best	4.708575	4.232750	3.771043	3.680268	2.808316
$CF_{27}$	Mean	−8.039649	−8.039721	−8.039795	−8.039843	−8.039849
	SD	1.857984 × 10^−4^	1.600838 × 10^−4^	6.850032 × 10^−5^	1.438728 × 10^−5^	1.628580 × 10^−6^
	Best	−8.039846	−8.039850	−8.039850	−8.039850	−8.039850
$CF_{28}$	Mean	0.114351	0.061762	0.066710	0.036362	0.0474869
	SD	0.096535	0.084652	0.076252	0.050605	0.048523
	Best	0.0001	0.0001	0.0001	0.0001	0.0001
$CF_{29}$	Mean	0.100030	0.082277	0.056272	0.024195	0.021766
	SD	0.056637	0.041447	0.047774	0.01818	0.009736
	Best	0.033410	0.0144334	0.010267	6.687262 × 10^−3^	6.000812 × 10^−3^
$CF_{30}$	Mean	0.446857	0.374940	0.266077	0.117528	0.132555
	SD	0.154262	0.168532	0.116847	0.041254	0.077086
	Best	0.123845	0.072605	0.079889	0.057579	4.514708 × 10^−4^
$CF_{31}$	Mean	−955.608723	−955.608723	−955.608723	−955.608723	−955.608723
	SD	6.283705 × 10^−13^	1.219156 × 10^−13^	1.219156 × 10^−13^	1.368971 × 10^−13^	1.850688 × 10^−13^
	Best	−955.608723	−955.608723	−955.608723	−955.608723	−955.608723

**Table 13:** Comparison of the GBFIO algorithm with other optimization algorithms.

		GBFIO	GWO	TLBO	DE	PSO
$CF_{1}$	Mean	0	0	1.294179 × 10^−81^	0.144674	4.966553 × 10^−2^
	SD	0	0	3.882538 × 10^−81^	0.234250	0.057479
	Best	0	0	9.879461 × 10^−174^	1.595589 × 10^−36^	8.364170 × 10^−3^
$CF_{2}$	Mean	1.218656 × 10^−287^	0	1.051319	5.177139 × 10^−44^	0.128009
	SD	0	0	3.153956	1.553142 × 10^−43^	5.629631 × 10^−2^
	Best	1.118360 × 10^−290^	0	7.386626 × 10^−90^	3.227326 × 10^−63^	0.025325
$CF_{3}$	Mean	0	3.192145 × 10^−105^	9.184483 × 10^−5^	166.611247	15.313370
	SD	0	9.5764341 × 10^−105^	2.755345 × 10^−4^	205.245492	10.381268
	Best	0	3.203688 × 10^−132^	1.279402 × 10^−19^	32.239259	7.060690
$CF_{4}$	Mean	6.351895 × 10^−267^	3.568757 × 10^−121^	1.337250 × 10^−25^	7.976398	3.098161
	SD	0	8.394264 × 10^−121^	4.009598 × 10^−25^	4.841287	0.960820
	Best	9.529171 × 10^−272^	6.329564 × 10^−127^	2.708885 × 10^−50^	2.436636	1.641832
$CF_{5}$	Mean	24.657226	25.000458	25.473331	82.627279	67.935807
	SD	0.344494	0.528751	1.014831	54.946209	50.904897
	Best	24.007749	24.142314	24.718418	27.056345	28.198511
$CF_{6}$	Mean	2.254566 × 10^−9^	7.538724 × 10^−2^	2.005264	1.574209 × 10^−3^	4.139476 × 10^−3^
	SD	2.433088 × 10^−9^	0.115157	0.278941	4.594092 × 10^−3^	2.617503 × 10^−3^
	Best	9.271979 × 10^−11^	1.580530 × 10^−7^	1.521674	6.430025 × 10^−26^	8.398675 × 10^−4^
$CF_{7}$	Mean	7.987476 × 10^−5^	1.731456 × 10^−4^	1.152873 × 10^−3^	2.018705 × 10^−3^	7.780638 × 10^−3^
	SD	2.957382 × 10^−5^	5.302466 × 10^−5^	7.507599 × 10^−4^	8.608555 × 10^−4^	3.925921 × 10^−3^
	Best	2.590416 × 10^−5^	8.293191 × 10^−5^	2.602298 × 10^−4^	7.265199 × 10^−4^	3.881654 × 10^−3^
$CF_{8}$	Mean	−7145.318974	−7765.884441	−5415.742450	−12,533.951559	−6618.143350
	SD	1888.294358	423.738554	817.169578	75.838506	715.725130
	Best	−9400.506153	−8317.183028	−7547.511021	−12,569.486618	−8027.302686
$CF_{9}$	Mean	1.534772 × 10^−13^	4.998223	147.657316	11.839696	28.485021
	SD	4.574788 × 10^−13^	6.107039	45.841691	9.225338	4.849056
	Best	0	0	62.506848	0.994959	19.965157
$CF_{10}$	Mean	4.440892 × 10^−15^	5.151435 × 10^−15^	13.552329	0.103796	2.803250
	SD	0	1.421085 × 10^−15^	7.694548	0.168057	0.420610
	Best	4.440892 × 10^−15^	4.440892 × 10^−15^	4.440892 × 10^−15^	4.440892 × 10^−15^	1.792227
$CF_{11}$	Mean	0	1.786773 × 10^−3^	9.573472 × 10^−3^	1.105455 × 10^−2^	8.989487 × 10^−2^
	SD	0	3.611421 × 10^−3^	2.220695 × 10^−3^	2.513669 × 10^−2^	4.414168 × 10^−2^
	Best	0	0	0	0	5.255478 × 10^−2^
$CF_{12}$	Mean	5.497947 × 10^−11^	2.470617 × 10^−8^	9.135967 × 10^−2^	4.669100 × 10^−2^	1.327286
	SD	4.249546 × 10^−11^	1.011952 × 10^−8^	2.430115 × 10^−2^	8.734069 × 10^−2^	1.247796
	Best	7.307790 × 10^−12^	1.197376 × 10^−8^	6.475929 × 10^−2^	1.570545 × 10^−32^	0.111829
$CF_{13}$	Mean	0.188401	6.580893 × 10^−2^	1.380241	2.362345	0.494989
	SD	0.153263	6.743503 × 10^−2^	0.239008	3.112145	0.598903
	Best	4.302022 × 10^−4^	3.078688 × 10^−7^	1.135953	5.242489 × 10^−10^	9.204132 × 10^−2^
$CF_{14}$	Mean	0.998003837794450	0.998003837794717	0.998003847208895	1.49108796655502	1.29581667593930
	SD	7.021667 × 10^−17^	2.011807 × 10^−13^	2.623667 × 10^−8^	1.479252	0.635439
	Best	0.998003837794450	0.998003837794483	0.998003837794450	0.998003837794450	0.998003837794450
$CF_{15}$	Mean	4.62448857988186 × 10^−4^	3.99054908144924 × 10^−4^	4.058168 × 10^−4^	6.984581 × 10^−4^	4.906235 × 10^−4^
	SD	2.745643 × 10^−4^	2.747062 × 10^−4^	2.736655 × 10^−4^	2.716092 × 10^−4^	3.662750 × 10^−4^
	Best	3.07485988525647 × 10^−4^	3.07485989805093 × 10^−4^	3.07662563819038 × 10^−4^	3.10985749911466 × 10^−4^	3.07485987805605 × 10^−4^
$CF_{16}$	Mean	−1.03162845348988	−1.03162845340201	−1.03162841182056	−1.03162845348988	−1.03162845348988
	SD	0	7.100377 × 10^−11^	9.916618 × 10^−8^	0	0
	Best	−1.03162845348988	−1.03162845348443	−1.03162845347704	−1.03162845348988	−1.03162845348988
$CF_{17}$	Mean	0.397887357729738	0.397887358687981	0.397887357729913	0.397887357729738	0.397887357729738
	SD	0	8.737636 × 10^−10^	4.454266 × 10^−13^	0	0
	Best	0.397887357729738	0.397887357729841	0.397887357729738	0.397887357729738	0.397887357729738
$CF_{18}$	Mean	2.99999999999992	3.00000001535671	3.00020912571569	2.99999999999992	2.99999999999992
	SD	5.063396 × 10^−16^	2.426698 × 10^−8^	5.874210 × 10^−4^	8.188600 × 10^−16^	0
	Best	2.99999999999992	3.00000000000157	2.99999999999992	2.99999999999992	2.99999999999992
$CF_{19}$	Mean	−3.86278214782076	−3.86278209251003	−3.86278214065809	−3.86278214782076	−3.86278214782076
	SD	8.881784 × 10^−16^	4.353004 × 10^−8^	1.224830 × 10^−8^	8.881784 × 10^−16^	8.881784 × 10^−16^
	Best	−3.86278214782076	−3.86278214667246	−3.86278214782076	−3.86278214782076	−3.86278214782076
$CF_{20}$	Mean	−3.191775	−3.202244	−3.198292	−3.200285	−3.189303
	SD	2.92343 × 10^−2^	2.459642 × 10^−2^	8.855541 × 10^−3^	7.268656 × 10^−6^	2.755499 × 10^−2^
	Best	−3.22219007647393	−3.22219006463194	−3.21360383726563	−3.20028745044100	−3.22219007647393
$CF_{21}$	Mean	−9.601349	−9.142709	−9.031591	−9.142714	−8.390441
	SD	1.516076	2.020970	1.175195	2.020971	2.767170
	Best	−10.1531996790582	−10.1531994004619	−10.1312343981895	−10.1531996790582	−10.1531996790582
$CF_{22}$	Mean	−10.242894	−9.339864	−9.674596	−9.871391	−8.872245
	SD	0.199721	2.126096	0.729454	1.594573	3.061341
	Best	−10.4029153367777	−10.4029149121881	−10.3983960082644	−10.4029153367777	−10.4029153367777
$CF_{23}$	Mean	−10.5364431534835	−10.5364381295375	−9.38574003225806	−10.5364431534835	−10.5364431534835
	SD	7.944109 × 10^−16^	4.450583 × 10^−6^	1.967776	0	1.375960 × 10^−15^
	Best	−10.5364431534835	−10.5364429443837	−10.5345358768162	−10.5364431534835	−10.5364431534835
$CF_{24}$	Mean	−106.764536749265	−106.764536670269	−106.764433302178	−106.764536749265	−106.764536749265
	SD	2.107810 × 10^−14^	1.062099 × 10^−7^	2.393181 × 10^−4^	1.421085 × 10^−14^	2.733512 × 10^−14^
	Best	−106.764536749265	−106.764536749056	−106.764536747555	−106.764536749265	−106.764536749265
$CF_{25}$	Mean	0.500000000000153	0.500000000000779	0.500000000000314	0.500000000000000	0.500000000000000
	SD	2.008861 × 10^−13^	1.627484 × 10^−12^	8.786089 × 10^−13^	0	0
	Best	0.500000000000005	0.500000000000001	0.500000000000000	0.500000000000000	0.500000000000000
$CF_{26}$	Mean	4.649871	2.701262	6.522691	3.648867	4.588877
	SD	0.800309	1.151474	1.031441	0.268596	0.723199
	Best	2.787816	0.931653	4.442681	3.038657	3.453176
$CF_{27}$	Mean	−8.03977154555592	−8.03934047668692	−8.03955534121555	−8.03983806190992	−8.03919251751744
	SD	1.185843 × 10^−4^	1.742601 × 10^−4^	2.361555 × 10^−4^	2.857435 × 10^−5^	6.104576 × 10^−4^
	Best	−8.039850	−8.039829	−8.039836	−8.039849	−8.039850
$CF_{28}$	Mean	6.097802 × 10^−2^	0.284577	0.190707	7.502779 × 10^−4^	2.107188 × 10^−4^
	SD	8.192341 × 10^−2^	0.113906	8.350326 × 10^−2^	5.310118 × 10^−4^	3.321565 × 10^−4^
	Best	0.0001	0.015002	0.044782	0.0001	0.0001
$CF_{29}$	Mean	0.257036	3.306600	7.702267	1.158201	3.942612
	SD	0.333940	4.187744	0.435606	1.187534	0.603973
	Best	1.453989 × 10^−2^	0.301483	6.647798	7.549659 × 10^−12^	2.797453
$CF_{30}$	Mean	0.336127	0.363262	0.603217	7.050466 × 10^−8^	0.245494
	SD	0.137485	0.182822	0.217843	1.619793 × 10^−7^	0.133411
	Best	5.559156 × 10^−2^	5.500235 × 10^−2^	0.313131	0	6.083741 × 10^−33^
$CF_{31}$	Mean	−955.608723	−950.418413	−955.608721	−954.570662	−891.830018
	SD	3.595093 × 10^−14^	5.190310	3.862493 × 10^−6^	3.114183	91.055620
	Best	−955.608722698531	−955.608722698531	−955.608722698531	−955.608722698531	−955.608722698531
Rank Based on Mean of the Results	Sum Rank	53	88	122	84	107
	Mean Rank	1.709677	2.838710	3.935484	2.709677	3.451613
	Total Rank	1	3	5	2	4
Rank Based on Best of the Results	Sum Rank	53	97	124	81	100
	Mean Rank	1.709677	3.129032	4	2.612903	3.225806
	Total Rank	1	3	5	2	4

**Table 14:** Unconstrained CEC2017 benchmark test functions.

No.	Function Name	$F^{*}$	Type
${CEC}_{01}$	S&R Bent Cigar Function	100	Unimodal
${CEC}_{02}$	S&R Sum of Different Power Function	200	
${CEC}_{03}$	S&R Zakharov Function	300	
${CEC}_{04}$	S&R Rosenbrock’s Function	400	Multimodal
${CEC}_{05}$	S&R Rastrigin’s Function	500	
${CEC}_{06}$	S&R Expanded Scaffer’s F6 Function	600	
${CEC}_{07}$	S&R Lunacek Bi_Rastrigin Function	700	
${CEC}_{08}$	S&R Non-Continuous Rastrigin’s Function	800	
${CEC}_{09}$	S&R Levy Function	900	
${CEC}_{10}$	S&R Schwefel’s Function	1000	
${CEC}_{11}$	${CEC}_{03}$, ${CEC}_{04}$, and ${CEC}_{05}$	1100	Hybrid
${CEC}_{12}$	${CEC}_{01}$, ${CEC}_{10}$, and S&R High-Conditioned Elliptic Function	1200	
${CEC}_{13}$	${CEC}_{01}$, ${CEC}_{04}$, and ${CEC}_{07}$	1300	
${CEC}_{14}$	${CEC}_{05}$, S&R High-Conditioned Elliptic, S&R Ackley, and S&R Expanded Scaffer’s F7 Functions	1400	
${CEC}_{15}$	${CEC}_{01}$, ${CEC}_{04}$, ${CEC}_{05}$, and S&R HGBat Function	1500	
${CEC}_{16}$	${CEC}_{04}$, ${CEC}_{06}$, ${CEC}_{10}$, and S&R HGBat Function	1600	
${CEC}_{17}$	${CEC}_{05}$, ${CEC}_{10}$, S&R Ackley, and S&R Expanded Griewank plus Rosenbrock Functions	1700	
${CEC}_{18}$	$CEC_{05}$, S&R High-Conditioned Elliptic, S&R Ackley, S&R HGBat, and S&R Discus Functions	1800	
${CEC}_{19}$	$CEC_{01}$, $CEC_{05}$, $CEC_{06}$, S&R Expanded Griewank Plus Rosenbrock, and S&R Weierstrass	1900	
${CEC}_{20}$	${CEC}_{05}$, S&R HappyCat, S&R Katsuura, S&R Ackley, S&R Schwefel, and S&R Expanded Scaffer’s F7 Functions	2000	
$CEC_{21}$	Rosenbrocks, High-Conditioned Elliptic, and Rastrigin’s Functions	2100	Composition
${CEC}_{22}$	Rastrigin’s, Griewank, and Modified Schwefel Functions	2200	
${CEC}_{23}$	Rosenbrocks, Ackley, Modified Schwefel, and Rastrigin’s Functions	2300	
${CEC}_{24}$	Ackley, High-Conditioned Elliptic, Griewank, and Rastrigin’s Functions	2400	
${CEC}_{25}$	Rastrigins, HappyCat, Ackley, Discus, and Rosenbrock’s Functions	2500	
${CEC}_{26}$	Expanded Scaffer’s F6, Modified Schwefel, Griewank, Rosenbrocks, and Rastrigin’s Functions	2600	
$CEC_{27}$	HappyCat, Rastrigins, Modified Schwefel, Bent Cigar, High-Conditioned Elliptic, and Expanded Scaffer’s F6 Functions	2700	
${CEC}_{28}$	Ackley, Griewank, Discus, Rosenbrocks, HappyCat, and Expanded Scaffer’s F6 Functions	2800	
${CEC}_{29}$	Expanded Scaffer’s F6, Ackley, Expanded Griewank plus Rosenbrock, Bent Cigar, Two HGBat, Two Rosenbrocks, Two Rastrigins, and Two Schwefel’s Functions	2900	
${CEC}_{30}$	Expanded Griewank Plus Rosenbrock, Weierstrass, Expanded Scaffer’s F6, High-Conditioned Elliptic, Ackley, Discus, Two HGBat, Two Rosenbrocks, Two Bent Cigar, and Three Rastrigin’s Functions	3000	

**Table 15:** Comparison based on shifted and rotated CEC2017 benchmark test functions.

		GBFIO	GWO	TLBO	DE	PSO	CPA	TSA	WOA
$C{EC}_{1}$	Mean	3.0427 × 10^+4^	5.2425 × 10^+7^	1.0787 × 10^+10^	3.2906 × 10^+6^	5.8511 × 10^+8^	5.5862 × 10^+4^	7.4852 × 10^+10^	1.7534 × 10^+8^
	SD	2.8249 × 10^+4^	6.8858 × 10^+7^	2.7243 × 10^+9^	7.0151 × 10^+6^	2.3514 × 10^+8^	8.6168 × 10^+4^	6.0034 × 10^+9^	2.7801 × 10^+8^
	Best	1.0006 × 10^+2^	2.2646 × 10^+6^	7.3081 × 10^+9^	1.0473 × 10^+2^	3.1247 × 10^+8^	1.3209 × 10^+2^	6.5858 × 10^+10^	1.2616 × 10^+2^
$CF_{2}$	Mean	1.3459 × 10^+16^	1.8283 × 10^+21^	1.0306 × 10^+30^	2.4881 × 10^+20^	6.5639 × 10^+16^	5.2363 × 10^+22^	3.3919 × 10^+38^	4.5171 × 10^+32^
	SD	3.7829 × 10^+16^	3.6753 × 10^+21^	1.6537 × 10^+30^	7.1219 × 10^+20^	1.9650 × 10^+17^	1.3691 × 10^+23^	8.5134 × 10^+38^	1.3551 × 10^+33^
	Best	3.0591 × 10^+8^	1.2396 × 10^+13^	2.3330 × 10^+26^	6.6152 × 10^+12^	1.0099 × 10^+10^	5.0366 × 10^+12^	4.0212 × 10^+36^	4.6817 × 10^+11^
$CF_{3}$	Mean	7.9853 × 10^+3^	1.2979 × 10^+3^	5.1089 × 10^+4^	1.1540 × 10^+4^	6.6428 × 10^+2^	1.7898 × 10^+4^	8.5956 × 10^+4^	6.6356 × 10^+2^
	SD	3.5092 × 10^+3^	1.0247 × 10^+3^	1.5404 × 10^+4^	3.5303 × 10^+3^	1.0050 × 10^+3^	7.8294 × 10^+3^	9.5798 × 10^+3^	4.6279 × 10^+2^
	Best	3.0623 × 10^+3^	3.5710 × 10^+2^	3.3715 × 10^+4^	7.1014 × 10^+3^	3.1513 × 10^+2^	7.0414 × 10^+3^	7.6401 × 10^+4^	3.0118 × 10^+2^
$CF_{4}$	Mean	8.4462 × 10^+3^	2.3143 × 10^+5^	4.3737 × 10^+8^	9.1427 × 10^+3^	5.9680 × 10^+5^	1.2831 × 10^+4^	8.5166 × 10^+9^	9.7340 × 10^+3^
	SD	9.1738 × 10^+3^	3.0453 × 10^+5^	2.1974 × 10^+8^	7.0608 × 10^+3^	1.4865 × 10^+6^	1.2426 × 10^+4^	1.7105 × 10^+9^	6.5563 × 10^+3^
	Best	6.1169 × 10^+2^	9.8791 × 10^+3^	1.5995 × 10^+8^	6.0642 × 10^+2^	7.8244 × 10^+2^	6.0885 × 10^+2^	6.7843 × 10^+9^	7.4653 × 10^+2^
$CF_{5}$	Mean	6.9705 × 10^+2^	9.8453 × 10^+2^	1.0757 × 10^+4^	6.5860 × 10^+2^	1.0612 × 10^+3^	6.9874 × 10^+2^	5.9355 × 10^+4^	4.8812 × 10^+3^
	SD	2.3280 × 10^+1^	2.8484 × 10^+2^	2.0250 × 10^+3^	8.8627	3.9530 × 10^+2^	3.6326 × 10^+1^	4.0418 × 10^+3^	9.4759 × 10^+2^
	Best	6.5335 × 10^+2^	6.0076 × 10^+2^	7.7623 × 10^+3^	6.4341 × 10^+2^	7.3606 × 10^+2^	6.0023 × 10^+2^	5.0626 × 10^+4^	3.4009 × 10^+3^
$CF_{6}$	Mean	6.1199 × 10^+2^	6.1076 × 10^+2^	6.1221 × 10^+2^	6.1204 × 10^+2^	6.1142 × 10^+2^	6.1229 × 10^+2^	6.1274 × 10^+2^	6.1225 × 10^+2^
	SD	0.2505	0.39161	0.2545	0.1725	0.4042	0.2568	0.1937	0.4033
	Best	6.1159 × 10^+2^	6.0998 × 10^+2^	6.1156 × 10^+2^	6.1181 × 10^+2^	6.1076 × 10^+2^	6.1156 × 10^+2^	6.1243 × 10^+2^	6.1157 × 10^+2^
$CF_{7}$	Mean	9.4332 × 10^+2^	9.7733 × 10^+2^	2.6965 × 10^+3^	8.9814 × 10^+2^	9.6824 × 10^+2^	9.4150 × 10^+2^	1.0159 × 10^+4^	3.3524 × 10^+3^
	SD	2.3829 × 10^+1^	3.1959 × 10^+1^	3.8496 × 10^+2^	9.4581	8.5816 × 10^+1^	8.8598	8.3824 × 10^+2^	9.7905 × 10^+2^
	Best	9.0639 × 10^+2^	9.4075 × 10^+2^	2.2887 × 10^+3^	8.7647 × 10^+2^	8.1326 × 10^+2^	9.2597 × 10^+2^	8.3332 × 10^+3^	2.3470 × 10^+3^
$CF_{8}$	Mean	1.0170 × 10^+3^	1.1686 × 10^+3^	2.0555 × 10^+4^	9.5874 × 10^+2^	2.5350 × 10^+3^	1.0173 × 10^+3^	9.0818 × 10^+4^	4.6828 × 10^+3^
	SD	1.6858 × 10^+1^	1.3060 × 10^+2^	3.0799 × 10^+3^	8.3148	7.6991 × 10^+2^	1.4184 × 10^+1^	9.1209 × 10^+3^	1.4188 × 10^+3^
	Best	9.8354 × 10^+2^	9.4557 × 10^+2^	1.5471 × 10^+4^	9.3628 × 10^+2^	1.1926 × 10^+3^	9.8525 × 10^+2^	7.2518 × 10^+4^	2.7142 × 10^+3^
$CF_{9}$	Mean	9.3282 × 10^+2^	1.0107 × 10^+3^	7.6405 × 10^+3^	9.0001 × 10^+2^	4.1650 × 10^+3^	9.1484 × 10^+2^	1.2363 × 10^+4^	6.0580 × 10^+3^
	SD	1.4807 × 10^+1^	7.4294 × 10^+1^	2.1172 × 10^+3^	3.7424 × 10^−2^	1.3628 × 10^+3^	9.7802	1.0314 × 10^+3^	1.8479 × 10^+3^
	Best	9.1759 × 10^+2^	9.0972 × 10^+2^	4.7597 × 10^+3^	9.0000 × 10^+2^	1.8404 × 10^+3^	9.0107 × 10^+2^	1.0089 × 10^+4^	3.5584 × 10^+3^
$CF_{10}$	Mean	1.1011 × 10^+4^	1.1079 × 10^+4^	1.1455 × 10^+4^	1.0722 × 10^+4^	1.1401 × 10^+4^	1.0961 × 10^+4^	1.2451 × 10^+4^	1.1813 × 10^+4^
	SD	1.8808 × 10^+2^	1.5078 × 10^+2^	2.3859 × 10^+2^	1.5673 × 10^+2^	1.2725 × 10^+2^	1.284 × 10^+2^	1.3989 × 10^+2^	4.1831 × 10^+2^
	Best	1.0590 × 10^+4^	1.0908 × 10^+4^	1.1136 × 10^+4^	1.0548 × 10^+4^	1.1195 × 10^+4^	1.0724 × 10^+4^	1.2189 × 10^+4^	1.1129 × 10^+4^
$CF_{11}$	Mean	1.7670 × 10^+3^	2.0221 × 10^+6^	3.1151 × 10^+9^	2.5291 × 10^+3^	1.1730 × 10^+7^	8.7801 × 10^+5^	4.0390 × 10^+10^	1.3320 × 10^+3^
	SD	6.7221 × 10^+2^	3.4853 × 10^+6^	8.2243 × 10^+8^	3.0633 × 10^+3^	2.0238 × 10^+7^	1.6759 × 10^+6^	4.4325 × 10^+9^	2.6880 × 10^+2^
	Best	1.1290 × 10^+3^	4.2580 × 10^+3^	1.5136 × 10^+9^	1.1365 × 10^+3^	1.0634 × 10^+4^	2.4347 × 10^+3^	3.2131 × 10^+10^	1.1290 × 10^+3^
$CF_{12}$	Mean	5.9288 × 10^+4^	9.7257 × 10^+7^	7.1215 × 10^+9^	1.6104 × 10^+4^	1.3794 × 10^+8^	5.7313 × 10^+6^	4.8662 × 10^+10^	1.3130 × 10^+9^
	SD	9.0807 × 10^+4^	1.3485 × 10^+8^	2.0435 × 10^+9^	2.5779 × 10^+3^	2.4600 × 10^+8^	1.1492 × 10^+7^	5.4498 × 10^+9^	3.1497 × 10^+9^
	Best	1.3827 × 10^+4^	4.7340 × 10^+6^	4.0387 × 10^+9^	1.3849 × 10^+4^	1.6120 × 10^+4^	1.4564 × 10^+4^	3.7038 × 10^+10^	1.3874 × 10^+4^
$CF_{13}$	Mean	8.3733 × 10^+4^	1.4049 × 10^+8^	1.4700 × 10^+10^	3.9304 × 10^+5^	5.8942 × 10^+8^	8.1524 × 10^+4^	8.5046 × 10^+10^	2.1004 × 10^+3^
	SD	2.6233 × 10^+4^	9.9947 × 10^+7^	3.6972 × 10^+9^	4.4340 × 10^+5^	2.9850 × 10^+8^	4.4050 × 10^+4^	1.3211 × 10^+10^	1.3268
	Best	3.3367 × 10^+4^	2.7440 × 10^+7^	1.0374 × 10^+10^	1.8847 × 10^+4^	8.4376 × 10^+7^	2.2178 × 10^+3^	5.9840 × 10^+10^	2.0984 × 10^+3^
$CF_{14}$	Mean	5.8750 × 10^+6^	1.5426 × 10^+7^	9.8656 × 10^+7^	6.3622 × 10^+6^	2.1211 × 10^+6^	9.4752 × 10^+6^	9.1320 × 10^+8^	9.3891 × 10^+5^
	SD	1.7853 × 10^+6^	8.1897 × 10^+6^	2.1676 × 10^+7^	2.8854 × 10^+6^	2.2163 × 10^+6^	1.0014 × 10^+7^	2.4638 × 10^+8^	6.2615 × 10^+5^
	Best	2.4620 × 10^+6^	5.0749 × 10^+6^	5.8264 × 10^+7^	2.2981 × 10^+6^	2.5551 × 10^+5^	1.5089 × 10^+6^	5.2074 × 10^+8^	2.9271 × 10^+5^
$CF_{15}$	Mean	8.2975 × 10^+4^	1.3341 × 10^+8^	1.0189 × 10^+10^	4.0740 × 10^+5^	3.0637 × 10^+8^	8.9142 × 10^+4^	7.6362 × 10^+10^	1.5357 × 10^+3^
	SD	3.9857 × 10^+4^	1.1885 × 10^+8^	2.4488 × 10^+9^	3.0513 × 10^+5^	3.0808 × 10^+8^	5.7921 × 10^+4^	3.9859 × 10^+9^	7.9048
	Best	1.5673 × 10^+3^	4.2886 × 10^+6^	6.8067 × 10^+9^	1.5325 × 10^+3^	3.2873 × 10^+7^	1.5468 × 10^+3^	6.9687 × 10^+9^	1.5299 × 10^+3^
$CF_{16}$	Mean	1.4487 × 10^+4^	2.5661 × 10^+5^	1.2361 × 10^+9^	5.8814 × 10^+4^	2.7922 × 10^+6^	3.6117 × 10^+4^	2.9758 × 10^+10^	3.2885 × 10^+5^
	SD	5.8891 × 10^+2^	4.0008 × 10^+5^	3.0577 × 10^+8^	7.6916 × 10^+4^	4.1507 × 10^+6^	6.4341 × 10^+4^	5.1031 × 10^+9^	8.6244 × 10^+5^
	Best	1.4194 × 10^+4^	1.4372 × 10^+4^	7.0565 × 10^+8^	1.4231 × 10^+4^	6.4617 × 10^+4^	1.4196 × 10^+4^	1.9522 × 10^+10^	1.4195 × 10^+4^
$CF_{17}$	Mean	1.4351 × 10^+4^	7.1725 × 10^+7^	1.6760 × 10^+14^	1.4279 × 10^+4^	9.7976 × 10^+8^	1.4331 × 10^+4^	5.4148 × 10^+16^	7.9218 × 10^+7^
	SD	2.0286 × 10^+1^	1.6587 × 10^+8^	1.3323 × 10^+14^	7.2173	2.2737 × 10^+9^	5.5655 × 10^+1^	1.4025 × 10^+16^	2.3744 × 10^+8^
	Best	1.4310 × 10^+4^	1.4474 × 10^+4^	2.4595 × 10^+13^	1.4270 × 10^+4^	4.7082 × 10^+6^	1.4297 × 10^+4^	3.2314 × 10^+16^	1.4310 × 10^+4^
$CF_{18}$	Mean	7.6719 × 10^+6^	1.2565 × 10^+7^	8.4578 × 10^+7^	1.7995 × 10^+7^	2.3636 × 10^+6^	1.1445 × 10^+7^	4.9030 × 10^+8^	3.9831 × 10^+7^
	SD	3.9782 × 10^+6^	5.1616 × 10^+6^	2.1103 × 10^+7^	1.3468 × 10^+7^	1.1146 × 10^+6^	6.8927 × 10^+6^	6.7935 × 10^+7^	1.8158 × 10^+7^
	Best	1.7226 × 10^+6^	5.8824 × 10^+6^	5.4018 × 10^+7^	6.7495 × 10^+6^	1.2019 × 10^+6^	5.2488 × 10^+6^	3.2780 × 10^+8^	1.4917 × 10^+7^
$CF_{19}$	Mean	4.7801 × 10^+3^	1.8763 × 10^+8^	5.7415 × 10^+13^	3.3488 × 10^+3^	8.8622 × 10^+8^	3.4119 × 10^+3^	2.5284 × 10^+16^	1.9531 × 10^+3^
	SD	1.3835 × 10^+3^	1.6251 × 10^+8^	8.8069 × 10^+13^	2.6690 × 10^+3^	1.3862 × 10^+9^	2.0317 × 10^+3^	1.2974 × 10^+16^	3.0686 × 10^+1^
	Best	1.9145 × 10^+3^	7.8634 × 10^+6^	3.5591 × 10^+12^	1.9074 × 10^+3^	2.7479 × 10^+7^	1.9135 × 10^+3^	7.0023 × 10^+15^	1.9237 × 10^+3^
$CF_{20}$	Mean	5.9888 × 10^+102^	1.6665 × 10^+84^	7.6404 × 10^+111^	1.5548 × 10^+19^	1.4427 × 10^+100^	1.0005 × 10^+91^	8.4624 × 10^+116^	7.1701 × 10^+109^
	SD	1.7792 × 10^+103^	4.9606 × 10^+84^	2.2625 × 10^+112^	4.6645 × 10^+19^	4.1334 × 10^+100^	2.2225 × 10^+91^	1.1522 × 10^+117^	1.9814 × 10^+110^
	Best	6.5653 × 10^+65^	1.4713 × 10^+63^	5.2358 × 10^+106^	1.4579 × 10^+4^	1.8321 × 10^+86^	2.6695 × 10^+85^	5.4253 × 10^+113^	4.2488 × 10^+103^
$CF_{21}$	Mean	2.1135 × 10^+3^	3.8159 × 10^+3^	9.5991 × 10^+3^	6.4467 × 10^+3^	3.3722 × 10^+3^	2.1853 × 10^+3^	5.8262 × 10^+4^	2.1123 × 10^+3^
	SD	5.9729	7.7727 × 10^+2^	1.8415 × 10^+3^	1.3007 × 10^+4^	5.9383 × 10^+2^	1.2686 × 10^+2^	1.5694 × 10^+4^	5.3425
	Best	2.1097 × 10^+3^	2.7056 × 10^+3^	7.0339 × 10^+3^	2.1097 × 10^+3^	2.4574 × 10^+3^	2.1098 × 10^+3^	3.1666 × 10^+4^	2.1097 × 10^+3^
$CF_{22}$	Mean	6.4059 × 10^+3^	6.4015 × 10^+3^	6.4382 × 10^+3^	6.4009 × 10^+3^	6.4497 × 10^+3^	6.4216 × 10^+3^	6.4082 × 10^+3^	6.4113 × 10^+3^
	SD	5.0426 × 10^−1^	1.3617	1.7149 × 10^+1^	2.1676	1.4282 × 10^+1^	2.6376 × 10^+1^	3.9606 × 10^−1^	6.2780
	Best	6.4047 × 10^+3^	6.3984 × 10^+3^	6.4068 × 10^+3^	6.3971 × 10^+3^	6.4235 × 10^+3^	6.4008 × 10^+3^	6.4073 × 10^+3^	6.4072 × 10^+3^
$CF_{23}$	Mean	5.4514 × 10^+3^	5.4583 × 10^+3^	5.8620 × 10^+3^	5.4702 × 10^+3^	5.6456 × 10^+3^	5.4948 × 10^+3^	6.5117 × 10^+3^	5.4470 × 10^+3^
	SD	3.5528	1.3281 × 10^+1^	1.3220 × 10^+2^	1.6258 × 10^+1^	2.6447 × 10^+2^	3.4469 × 10^+1^	1.6655 × 10^+2^	7.6078
	Best	5.4496 × 10^+3^	5.4496 × 10^+3^	5.7378 × 10^+3^	5.4496 × 10^+3^	5.4781 × 10^+3^	5.4567 × 10^+3^	6.2514 × 10^+3^	5.4445 × 10^+3^
$CF_{24}$	Mean	2.4049 × 10^+3^	3.7702 × 10^+3^	8.1763 × 10^+3^	2.4003 × 10^+3^	4.4037 × 10^+3^	9.8171 × 10^+3^	4.4376 × 10^+4^	2.4112 × 10^+3^
	SD	3.5938	7.0818 × 10^+2^	1.9869 × 10^+3^	9.3908 × 10^−1^	1.6095 × 10^+3^	2.2245 × 10^+4^	1.2943 × 10^+4^	4.8607
	Best	2.4000 × 10^+3^	2.9135 × 10^+3^	5.8856 × 10^+3^	2.4000 × 10^+3^	2.5242 × 10^+3^	2.4000 × 10^+3^	2.6345 × 10^+4^	2.4047 × 10^+3^
$CF_{25}$	Mean	2.5115 × 10^+3^	2.5259 × 10^+3^	2.8632 × 10^+3^	2.5346 × 10^+3^	2.6545 × 10^+3^	2.5449 × 10^+3^	3.6612 × 10^+3^	2.5209 × 10^+3^
	SD	1.3832 × 10^+1^	2.1977 × 10^+1^	5.0626 × 10^+1^	6.1919 × 10^+1^	9.3843 × 10^+1^	2.5118 × 10^+1^	1.4584 × 10^+2^	1.5459 × 10^+1^
	Best	2.5069 × 10^+3^	2.5069 × 10^+3^	2.7889 × 10^+3^	2.5069 × 10^+3^	2.5790 × 10^+3^	2.5071 × 10^+3^	3.4479 × 10^+3^	2.5069 × 10^+3^
$CF_{26}$	Mean	5.1221 × 10^+3^	5.1271 × 10^+3^	5.4637 × 10^+3^	5.1439 × 10^+3^	5.1698 × 10^+3^	5.1538 × 10^+3^	5.9227 × 10^+3^	5.1204 × 10^+3^
	SD	5.2682	1.5539 × 10^+1^	6.5653 × 10^+1^	4.3448 × 10^+1^	4.2873 × 10^+1^	1.8060 × 10^+1^	1.9952 × 10^+2^	1.0649 × 10^+1^
	Best	5.1197 × 10^+3^	5.1197 × 10^+3^	5.3657 × 10^+3^	5.1197 × 10^+3^	5.1228 × 10^+3^	5.1211 × 10^+3^	5.6535 × 10^+3^	5.1150 × 10^+3^
$CF_{27}$	Mean	4.7961 × 10^+3^	5.0083 × 10^+3^	1.3037 × 10^+6^	4.8113 × 10^+3^	5.2446 × 10^+3^	4.8678 × 10^+3^	2.3729 × 10^+6^	4.7963 × 10^+3^
	SD	5.2592 × 10^−1^	1.9426 × 10^+2^	2.6522 × 10^+5^	4.6254 × 10^+1^	3.0010 × 10^+2^	1.3515 × 10^+2^	2.5672 × 10^+5^	6.7444 × 10^−1^
	Best	4.7958 × 10^+3^	4.7968 × 10^+3^	9.5446 × 10^+5^	4.7958 × 10^+3^	4.9123 × 10^+3^	4.7959 × 10^+3^	2.0097 × 10^+6^	4.7958 × 10^+3^
$CF_{28}$	Mean	2.8057 × 10^+3^	2.8141 × 10^+3^	3.1012 × 10^+3^	2.8232 × 10^+3^	2.8946 × 10^+3^	2.8122 × 10^+3^	3.7470 × 10^+3^	2.8057 × 10^+3^
	SD	2.4908 × 10^−13^	1.0768 × 10^+1^	6.2779 × 10^+1^	1.8079 × 10^+1^	1.2846 × 10^+2^	1.3537 × 10^+1^	1.8942 × 10^+2^	2.0667 × 10^−8^
	Best	2.8057 × 10^+3^	2.8057 × 10^+3^	3.0038 × 10^+3^	2.8057 × 10^+3^	2.8143 × 10^+3^	2.8057 × 10^+3^	3.5139 × 10^+3^	2.8057 × 10^+3^
$CF_{29}$	Mean	4.9999 × 10^+3^	5.0411 × 10^+3^	5.2807 × 10^+5^	8.6630 × 10^+3^	6.3626 × 10^+3^	2.4707 × 10^+7^	1.1877 × 10^+6^	4.9999 × 10^+3^
	SD	8.1348 × 10^−13^	5.4386 × 10^+1^	4.0488 × 10^+4^	1.0984 × 10^+4^	1.5954 × 10^+3^	6.0227 × 10^+7^	8.1164 × 10^+4^	1.9679 × 10^−7^
	Best	4.9999 × 10^+3^	5.0005 × 10^+3^	4.8404 × 10^+5^	4.9999 × 10^+3^	5.0830 × 10^+3^	5.0104 × 10^+3^	1.0129 × 10^+6^	4.9999 × 10^+3^
$CF_{30}$	Mean	3.0022 × 10^+3^	3.0024 × 10^+3^	1.0060 × 10^+6^	7.1836 × 10^+5^	3.2555 × 10^+3^	9.2082 × 10^+6^	2.0871 × 10^+6^	3.0023 × 10^+3^
	SD	9.2714 × 10^−4^	3.0809 × 10^−2^	2.6791 × 10^+5^	2.1461 × 10^+6^	2.4063 × 10^+2^	2.7196 × 10^+7^	3.0555 × 10^+5^	3.7938 × 10^−2^
	Best	3.0022 × 10^+3^	3.0023 × 10^+3^	7.1959 × 10^+5^	3.0022 × 10^+3^	3.0712 × 10^+3^	3.1290 × 10^+3^	1.6273 × 10^+6^	3.0023 × 10^+3^
Rank Based on Mean of the Results	Mean Rank	2.233	3.933	6.733	2.933	4.733	4	7.8	3.633
	Total Rank	1	4	7	2	6	5	8	3
Rank Based on Best of the Results	Mean Rank	2.533	4.1	6.767	2.833	4.900	3.533	7.967	3.367
	Total Rank	1	5	7	2	6	4	8	3

**Table 16:** Performance of the GBFIO and other algorithms on the TCSD problem.

Algorithm		GBFIO	GWO	TLBO	DE	PSO
Variables	$x_{1}$	0.051733	0.051131	0.051559	0.051689	0.051691
	$x_{2}$	0.357782	0.343415	0.353596	0.356717	0.356762
	$x_{3}$	11.226838	12.119772	11.475790	11.289034	11.28640
Constraints	$g_{1}$	−2.220446 × 10^−16^	−1.137609 × 10^−4^	−7.716146 × 10^−5^	0	−2.220446 × 10^−16^
	$g_{2}$	0	−2.930045 × 10^−5^	−3.314506 × 10^−5^	0	0
	$g_{3}$	−4.049367	−4.047656	−4.047007	−4.053783	−4.053872
	$g_{4}$	−0.729275	−0.729614	−0.729896	−0.727730	−0.727698
Optimum objective function		0.012665	0.012667	0.012667	0.012665	0.012665

**Table 17:** Statistical results of the GBFIO algorithm in comparison with other algorithms on the TCSD problem.

Algorithm	GBFIO	GWO	TLBO	DE	PSO
Best	0.012665	0.012667	0.012667	0.012665	0.012665
Mean	0.012668	0.012702	0.012698	0.012744	0.012705
Worst	0.012676	0.012725	0.012763	0.013090	0.013193
SD	3.05638 × 10^−6^	2.07286 × 10^−5^	2.44736 × 10^−5^	1.24856 × 10^−4^	1.14067 × 10^−4^

**Table 18:** Performance of the GBFIO and other algorithms on the WBD problem.

Algorithm		GBFIO	GWO	TLBO	DE	PSO
Variables	$x_{1}$	0.203662	0.203663	0.203726	0.203671	0.203669
	$x_{2}$	2.657678	2.656573	2.662157	2.659399	2.659002
	$x_{3}$	9.474038	9.475445	9.467296	9.472102	9.472551
	$x_{4}$	0.203662	0.203667	0.203735	0.203671	0.203669
Constraints	$g_{1}$	−0.000427	−0.640565	−0.173804	0	0
	$g_{2}$	−2429.184048	−2437.957204	−2399.718172	−2419.093017	−2421.428131
	$g_{3}$	−0.241422	−0.241425	−0.241413	−0.241419	−0.241420
	$g_{4}$	−6.790706 × 10^−9^	−3.767273 × 10^−7^	−9.238739 × 10^−6^	0	0
	$g_{5}$	−0.009883	−0.960555	−3.702637	−0.004736	−9.094947 × 10^−13^
	$g_{6}$	−0.078662	−0.078663	−0.078726	−0.078671	−0.078669
	$g_{7}$	−3.407872	−3.407712	−3.407979	−3.407958	−3.407938
Optimum objective function		1.668085	1.668196	1.668231	1.668085	1.668085

**Table 19:** Statistical results of the GBFIO algorithm in comparison to other algorithms on the WBD problem.

Algorithm	GBFIO	GWO	TLBO	DE	PSO
Best	1.668085	1.668196	1.668231	1.668085	1.668085
Mean	1.668139	1.668613	1.673312	1.750973	1.670535
Worst	1.668261	1.670510	1.714264	2.277017	1.692367
SD	4.02387 × 10^−5^	0.000491	0.011483	0.139742	0.007278

**Table 20:** Performance of the GBFIO and other algorithms on the PVD problem.

Algorithm		GBFIO	GWO	TLBO	DE	PSO
Variables	$x_{1}$	0.778171	0.778334	0.778177	0.799488	0.778169
	$x_{2}$	0.384650	0.384867	0.384655	0.396177	0.384649
	$x_{3}$	40.319716	40.32289	40.31969	41.42425	40.31962
	$x_{4}$	199.998644	199.9564541	200	185.17393	200
Constraints	$g_{1}$	−3.474887 × 10^−12^	−1.026458 × 10^−4^	−6.906605 × 10^−6^	0	−1.110223 × 10^−16^
	$g_{2}$	−7.984169 × 10^−13^	−1.867439 × 10^−4^	−5.317988 × 10^−6^	−9.897130 × 10^−4^	0
	$g_{3}$	−8.302741 × 10^−7^	−10.026897	−4.945010	0	−4.656613 × 10^−10^
	$g_{4}$	−40.001356	−40.043546	−40	−54.826069	−40
Optimum objective function		5885.335987	5886.766716	5885.421274	5925.80197	5885.332774

**Table 21:** Statistical results of the GBFIO algorithm in comparison to other algorithms on the PVD problem.

Algorithm	GBFIO	GWO	TLBO	DE	PSO
Best	5885.335987	5886.766716	5885.421274	5925.80197	5885.332774
Mean	5885.729959	5906.428534	5957.584751	6268.301804	5987.870488
Worst	5887.656004	6159.109518	6413.063728	7107.412167	6409.398064
SD	0.504172297	58.24275335	131.2308551	294.4901147	165.3137231

**Table 22:** Performance of the GBFIO and other algorithms on the SRD problem.

Algorithm		GBFIO	GWO	TLBO	DE	PSO
Variables	$x_{1}$	3.5	3.501233	3.5	3.5	3.5
	$x_{2}$	0.7	0.7	0.7	0.7	0.7
	$x_{3}$	17	17	17	17	17
	$x_{4}$	7.3	7.307395699	7.3	7.3	7.3
	$x_{5}$	7.715319911	7.787380246	7.715385227	7.715319911	8.3
	$x_{6}$	3.350215	3.350762	3.350221	3.350215	3.350215
	$x_{7}$	5.286654	5.287165	5.28666	5.286654	5.286859
Constraints	$g_{1}$	−7.391528 × 10^−2^	−7.424131 × 10^−2^	−7.391585 × 10^−2^	−7.391528 × 10^−2^	−7.391528 × 10^−2^
	$g_{2}$	−0.197999	−0.198281	−0.197999	−0.197999	−0.197999
	$g_{3}$	−0.499172	−0.497977	−0.499176	−0.499172	−0.499172
	$g_{4}$	−0.904644	−0.901985	−0.904642	−0.904644	−0.881299
	$g_{5}$	−7.771561 × 10^−16^	−4.776360 × 10^−4^	−5.500447 × 10^−6^	−7.771561 × 10^−16^	−2.220446 × 10^−16^
	$g_{6}$	0	−2.755579 × 10^−4^	−3.228823 × 10^−6^	0	−2.220446 × 10^−16^
	$g_{7}$	−0.702500	−0.702500	−0.702500	−0.702500	−0.702500
	$g_{8}$	−3.330669 × 10^−16^	−3.520475 × 10^−4^	−6.164804 × 10^−7^	−2.220446 × 10^−16^	−2.220446 × 10^−16^
	$g_{9}$	−0.583333	−0.583187	−0.583333	−0.583333	−0.583333
	$g_{10}$	−5.132575 × 10^−2^	−5.217353 × 10^−2^	−5.132449 × 10^−2^	−5.132575 × 10^−2^	−5.132575 × 10^−2^
	$g_{11}$	−7.771561 × 10^−16^	−9.181416 × 10^−3^	−7.651281 × 10^−6^	−9.992007 × 10^−16^	−7.041622 × 10^−2^
Optimum objective function		2994.471066	2997.066108	2994.478541	2994.471066	3007.436552

**Table 23:** Statistical results of the GBFIO algorithm in comparison to other algorithms on the SRD problem.

Algorithm	GBFIO	GWO	TLBO	DE	PSO
Best	2994.471066	2997.06611	2994.478541	2994.471066	3007.436552
Mean	2994.471066	3001.723014	3002.476476	2995.286717	3049.074191
Worst	2994.471066	3009.972106	3037.194378	2999.867867	3180.009085
SD	3.02268 × 10^−10^	3.759029713	11.22155472	1.496503751	34.49904775

## Data Availability

The original contributions presented in the study are included in the article; further inquiries can be directed to the first and corresponding authors. The Matlab source code for the GBFIO algorithm is provided in the Appendix A and Supplementary File.

## Supplementary Materials

The following supporting information can be downloaded at: https://www.mdpi.com/article/10.3390/biomimetics10060379/s1.

## Appendix A

In this appendix, the Matlab source code of the GBFIO algorithm for the first objective function is presented as follows:

clc clear all pack %%%%%%%%%% n is dimension of objective function n = 30; %%%%%%%%%% Define the Range of Variables Xmax = ones(1,n) ×100; Xmin = ones(1,n)*(−100); %%%%%%%%%% n is the Population Size N = 150; %%%%%%%%%% Maximum Numbe od Iteration Iter_max = 500; %%%%%%%%%% Define the GBFIO prameters f = 2.5; eta = 1.25; Zeta = 0.3; fishing_number = 25; %%%%%%%%%% Generate the Initial Populations for i = 1:N Ipop(i,:) = rand(1,n).*(Xmax − Xmin) + Xmin; cost(i,:) = sum(Ipop(i,:).^2); end %%%%%%%%%% Sort the Initial Populations Based on Cost Function Ipop_mix = [Ipop,cost]; Ipop_sort = sortrows(Ipop_mix,n + 1); %%%%%%%%%% Specify the Fish, Honey, Body, Shell, and Plants Fish = Ipop_sort(1,:); Honey = Ipop_sort(2,:); Body = Ipop_sort(3,:); Shell = Ipop_sort(4,:); Plants = Ipop_sort(5,:); %%%%%%%%%% Start the Iterationa for Updating Populations for iter = 1:Iter_max for i = 1:N %%%%%%%%%% Start the Searching Phase r1 = rand(1,n); deltax_fish = (2.*r1.*( (r1).^0.5)).*(Fish(1,[1:n]) − Ipop(i,:)); deltax_honey = (r1.*((r1).^0.5)).*(Honey(1,[1:n]) − Ipop(i,:)); deltax_body = (0.5.*r1.*((r1).^0.5)).*(Body(1,[1:n]) − Ipop(i,:)); deltax_shell = (0.25.*r1.*((r1).^0.5)).*(Shell(1,[1:n]) − Ipop(i,:)); deltax_plants = (0.125.*r1.*((r1).^0.5)).*(Plants(1,[1:n]) − Ipop(i,:)); Xnew_search = Ipop(i,:) + deltax_fish + deltax_honey + deltax_body + deltax_shell + deltax_plants; Xnew_search = min(Xnew_search,Xmax); Xnew_search = max(Xnew_search,Xmin); cost_S = sum(Xnew_search.^2); %%%%%%%%%% Update the Population if cost_S < cost(i,1) Ipop(i,:) = Xnew_search; cost(i,1) = cost_S; end %%%%%%%%%% End the Searching Phase %%%%%%%%%% Start the Hunting Phase %%%%% Start the Hunting Phase: Bear Hunting Ipop_mix = [Ipop,cost]; Ipop_sort = sortrows(Ipop_mix,n + 1); Prey = Ipop_sort(1,:); A = (f*(1-iter/Iter_max)).*(2.*rand(1,n) − 1); D = abs(((2.*rand(1,n)).*Ipop(i,:))-(rand(1,n).*Prey(1,[1:n]))); X_hunt = Ipop(i,:)-A.*D; X_hunt = min(X_hunt,Xmax); X_hunt = max(X_hunt,Xmin); cost_hunt = sum(X_hunt.^2); %%%%% Start the Hunting Phase: Coyote Hunting L = randperm(N); LL = find(L~ = i); LLL = L(LL); J1 = LL(1); J2 = LL(2); J3 = LL(3); I_child = [Ipop(J1,:),cost(J1,1);Ipop(J2,:),cost(J2,1);Ipop(J3,:),cost(J3,1)]; I_chsort = sortrows(I_child,n + 1); A1 = (eta*(1-iter/Iter_max)).*(2.*rand(1,n) − 1); A2 = (eta*(1-iter/Iter_max)).*(2.*rand(1,n) − 1); D1 = abs(((2.*rand(1,n)).*I_chsort(2,[1:n])) − (rand(1,n).*I_chsort(1,[1:n]))); D2 = abs(((2.*rand(1,n)).*I_chsort(3,[1:n])) − (rand(1,n).*I_chsort(1,[1:n]))); X_ch = Ipop(i,:) − (A1.*D1 + A2.*D2); X_ch = min(X_ch,Xmax); X_ch = max(X_ch,Xmin); cost_ch = sum(X_ch.^2); %%%%%%%%%% Update the Population G = rand(1,1); if G < 0.75 if cost_hunt < cost(i,1) Ipop(i,:) = X_hunt; cost(i,1) = cost_hunt; end else if cost_ch < cost(i,1) Ipop(i,:) = X_ch; cost(i,1) = cost_ch; end end %%%%%%%%%% End the Hunting Phase %%%%%%%%%% Start the Fishing Phase for kk = 1:fishing_number Z = Zeta*(1 − iter/Iter_max)*cos(2*pi*rand(1,1)); X_fish = (1 + Z).*Ipop(i,:); X_fish = min(X_fish,Xmax); X_fish = max(X_fish,Xmin); cost_fishing = sum(X_fish.^2); %%%%%%%%%% Update the Population if cost_fishing < cost(i,1) Ipop(i,:) = X_fish; cost(i,1) = cost_fishing; end end %%%%%%%%%% Specify the Fish, Honey, Body, Shell, and Plants for Ipop_mix = [Ipop,cost]; Ipop_sort = sortrows(Ipop_mix,n + 1); Fish = Ipop_sort(1,:); Honey = Ipop_sort(2,:); Body = Ipop_sort(3,:); Shell = Ipop_sort(4,:); Plants = Ipop_sort(5,:); end %%%%%%%%%% Specify the Parameters of Convergence Curve Convergence_Curve(iter,1) = iter; Convergence_Curve(iter,2) = Ipop_sort(1,n + 1); end %%%%%%%%%% Print the Best Soultion Best = Ipop_sort(1,:) plot(Convergence_Curve(:,1),Convergence_Curve(:,2))
